# Cortical matrix remodeling as a hallmark of relapsing–remitting neuroinflammation in MR elastography and quantitative MRI

**DOI:** 10.1007/s00401-023-02658-x

**Published:** 2024-01-04

**Authors:** Rafaela V. Silva, Anna S. Morr, Helge Herthum, Stefan P. Koch, Susanne Mueller, Clara S. Batzdorf, Gergely Bertalan, Tom Meyer, Heiko Tzschätzsch, Anja A. Kühl, Philipp Boehm-Sturm, Jürgen Braun, Michael Scheel, Friedemann Paul, Carmen Infante-Duarte, Ingolf Sack

**Affiliations:** 1 Experimental and Clinical Research Center, a cooperation between the Max Delbrück Center for Molecular Medicine in the Helmholtz Association and Charité - Universitätsmedizin Berlin, Berlin, Germany; 2 Charité - Universitätsmedizin Berlin, Corporate Member of Freie Universität Berlin and Humboldt-Universität zu Berlin, ECRC - Experimental and Clinical Research Center, Berlin, Germany; 3 Max Delbrück Center for Molecular Medicine in the Helmholtz Association (MDC), Berlin, Germany; 4 Charité - Universitätsmedizin Berlin, Einstein Center for Neurosciences Berlin, Berlin, Germany; 5 Charité - Universitätsmedizin Berlin, Department of Radiology, Corporate Member of Freie Universität Berlin and Humboldt-Universität zu Berlin, Berlin, Germany; 6 Charité - Universitätsmedizin Berlin, Corporate Member of Freie Universität Berlin and Humboldt-Universität zu Berlin, Center for Advanced Neuroimaging, Berlin, Germany; 7 Charité - Universitätsmedizin Berlin, Corporate Member of Freie Universität Berlin and Humboldt-Universität zu Berlin, Department of Experimental Neurology and Center for Stroke Research Berlin, Berlin, Germany; 8 Charité-Universitätsmedizin Berlin, NeuroCure Cluster of Excellence and Charité Core Facility 7T Experimental MRI, Berlin, Germany; 9 Charité - Universitätsmedizin Berlin, Corporate Member of Freie Universität Berlin and Humboldt-Universität zu Berlin, iPATH.Berlin, Berlin, Germany; 10 Charité - Universitätsmedizin Berlin, Corporate Member of Freie Universität Berlin and Humboldt-Universität zu Berlin, Institute of Medical Informatics, Berlin, Germany; 11 Charité - Universitätsmedizin Berlin Corporate, Member of Freie Universität Berlin, Humboldt-Universität zu Berlin, Berlin Institute of Health, NeuroCure Clinical Research Center, Berlin, Germany; 12 Charité - Universitätsmedizin Berlin, Corporate Member of Freie Universität Berlin, Humboldt-Universität zu Berlin, Department of Neuroradiology, Berlin, Germany

**Keywords:** Magnetic resonance elastography, Tomoelastography, Multiple sclerosis, Experimental autoimmune encephalomyelitis, Cerebral cortex, Perineuronal nets

## Abstract

Multiple sclerosis (MS) is a chronic neuroinflammatory disease that involves both white and gray matter. Although gray matter damage is a major contributor to disability in MS patients, conventional clinical magnetic resonance imaging (MRI) fails to accurately detect gray matter pathology and establish a clear correlation with clinical symptoms. Using magnetic resonance elastography (MRE), we previously reported global brain softening in MS and experimental autoimmune encephalomyelitis (EAE). However, it needs to be established if changes of the spatiotemporal patterns of brain tissue mechanics constitute a marker of neuroinflammation. Here, we use advanced multifrequency MRE with tomoelastography postprocessing to investigate longitudinal and regional inflammation-induced tissue changes in EAE and in a small group of MS patients. Surprisingly, we found reversible softening in synchrony with the EAE disease course predominantly in the cortex of the mouse brain. This cortical softening was associated neither with a shift of tissue water compartments as quantified by T2-mapping and diffusion-weighted MRI, nor with leukocyte infiltration as seen by histopathology. Instead, cortical softening correlated with transient structural remodeling of perineuronal nets (PNNs), which involved abnormal chondroitin sulfate expression and microgliosis. These mechanisms also appear to be critical in humans with MS, where tomoelastography for the first time demonstrated marked cortical softening. Taken together, our study shows that neuroinflammation (i) critically affects the integrity of PNNs in cortical brain tissue, in a reversible process that correlates with disease disability in EAE, (ii) reduces the mechanical integrity of brain tissue rather than leading to water accumulation, and (iii) shows similar spatial patterns in humans and mice. These results raise the prospect of leveraging MRE and quantitative MRI for MS staging and monitoring treatment in affected patients.

**Supplementary Information:**

The online version contains supplementary material available at 10.1007/s00401-023-02658-x.

## Introduction

Multiple sclerosis (MS) is a chronic autoimmune disease of the central nervous system (CNS) with a rising incidence [72]. During MS, myelin-reactive cells infiltrate the CNS, leading to formation of multifocal lesions in both gray and white matter with subsequent demyelination and neurodegeneration [68]. There is accumulating evidence that not only white matter lesions but also gray matter pathology contributes significantly to disease disability in MS patients [31, 66]. Cognitive impairment and clinical disability have been shown to better correlate with the degree of cortical pathology than white matter damage [9, 49]. However, cortical pathology is difficult to quantify noninvasively by conventional clinical magnetic resonance imaging (MRI) with T1-weighted and T2-weighted pulse sequences because measurement of cortical thickness and the counting of cortical lesions are prone to partial volume effects [41]. Furthermore, the development of quantitative imaging markers that reflect a patient’s clinical presentation, therapeutic response, and predicted course of MS is hampered by the fact that the structural changes underlying cortical MS pathology are still incompletely understood [32].

Consequently, alternative MRI tools based on biophysical contrast mechanisms have been developed to leverage MRI for monitoring MS patients without using contrast agents [12, 74]. Among biophysical tissue properties, stiffness is a particularly interesting parameter because it scales with the hierarchy of mechanical networks in the brain, from individual neurons to gross anatomy [8, 39]. This scaling property makes stiffness a suitable quantitative imaging marker over wide dynamic ranges and spatial resolutions in both humans and mouse models [3, 46]. Brain stiffness can be measured noninvasively using acoustic vibrations that stimulate intracranial shear waves, as done in magnetic resonance elastography (MRE) [20, 35, 55, 56]. Cerebral MRE [7, 76] has been used to study changes in the mechanical consistency of the brain associated with both various physiological processes [27, 57] and neurological disorders [15, 38, 47, 65, 67, 75]. In MS patients, MRE revealed disseminated softening of the entire brain [75]. The observed softening was moderate in early disease [15] and pronounced in chronic MS [67]. Correspondingly, MRE in a mouse model of MS, experimental autoimmune encephalomyelitis (EAE), showed brain softening to be associated with demyelination [61], neuroinflammation [44, 54], and matrix remodeling [2, 63, 73]. However, in the past, low anatomical detail resolution of cerebral MRE hampered in-depth mechanistic studies in correlation with histopathology and in relation to regional segmentation of brains affected by EAE or MS [26, 54]. Here, we address these aspects using cerebral tomoelastography—an advanced multifrequency MRE technique for pixel-resolved quantification of brain stiffness in mice [4] and humans [25]. To investigate the underlying mechanisms of stiffness changes and to elucidate the contribution of the cortex, we combined tomoelastography with quantitative MRI and histopathological analysis in the EAE mouse model. Based on recent insights into EAE-induced extracellular matrix (ECM) remodeling [2, 53, 73], we hypothesize that the ECM of neuronal structures, organized in part in perineuronal nets (PNNs), critically determines tissue mechanical parameters in the brain and provides a contrast mechanism for early detection of neuroinflammation-related tissue damage using MRE. PNNs provide a dense, well-organized aggregate of chondroitin sulfate (CS), hyaluronic acid, tenascin, and link proteins [37] that surrounds somata and dendrites of neurons and interacts with other ECM components [13]. Thus, any structural alteration of PNNs may deteriorate the mechanical scaffold of perineuronal ECM, potentially leading to altered water binding properties and overall tissue softening [48]. The aim of this study is twofold: first, to decipher the mechanisms underlying in vivo brain softening in neuroinflammation at different stages of disease activity, and, second, to analyze the spatiotemporal patterns of brain softening with the potential for clinical translation as imaging marker and verify them in a small group of MS patients using the same technique previously applied in mice.

## Materials and methods

### Animals and EAE induction

Active EAE was induced in three groups of 9–12-week-old female SJL mice (*n* = 35 total [group 1 = 5, group 2 = 14, group 3 = 16]; Janvier, SAS, France). The mice were immunized with a subcutaneous injection of an emulsion containing 250 µg of the myelin peptide (PLP) 139–151, 100 µl complete Freund’s adjuvant (Thermo Fisher Scientific, USA), and 800 µg Mycobacterium tuberculosis H37Ra (Difco, USA) as previously described [2]. After immunization, mice were daily monitored for signs of disease, scored as follows: 0—no sign, 0.5—tail paresis, 1—tail plegia or tail paresis and righting reflex weakness; 1.5—tail plegia and righting reflex weakness; 2—hind limb paresis; 2.5—hind limb paresis or partial hind limb paralysis; 3—paraplegia; 4—paraplegia with forelimb weakness or paralysis; 5—moribund or dead animal. Fifteen mice from the three groups (group 1 = 5, group 2 = 10) were imaged longitudinally, and 20 mice (group 1 = 4, group 2 = 16) were imaged cross-sectionally with collection of tissue at peak (*n* = 10), remission (*n* = 7), and relapse (*n* = 3). The final number of mice in the remission and relapse groups additionally included animals investigated longitudinally, resulting in 11 mice in the remission group and 6 mice and the relapse group. In addition, 8 naïve female SJL mice were included in the study for additional MRE and histological analysis.

### Patients and healthy volunteers

A total of 12 patients (9 female, 3 male; age range 31–45 years, mean ± SD = 38 ± 5 years) with stable relapsing–remitting MS and 14 healthy volunteers (7 female, 7 male; age range 27–57 years, mean + SD = 38 ± 9 years) were included in this study. All patients were on pharmacological treatment with standard immunomodulatory agents (glatiramer acetate, dimethyl fumarate, teriflunomide, fingolimod or natalizumab), while healthy subjects had no history of neurological events.

### In vivo MRI and MRE in EAE

**Fig. 1:**
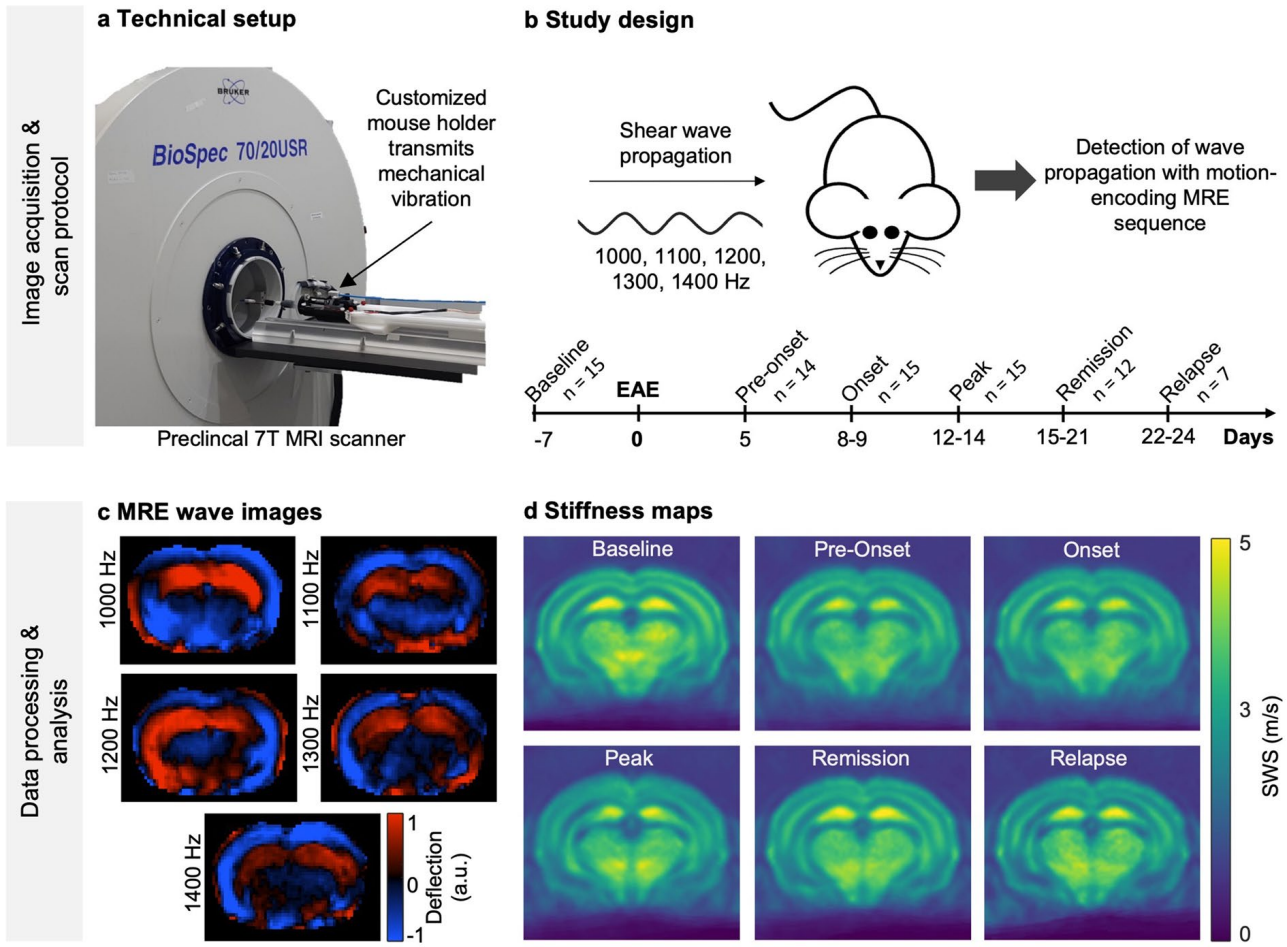
Experimental design and in vivo MRE in the EAE mouse model. **a** Technical setup. A customized animal holder inserted into a 7-T preclinical MRI scanner generates mechanical waves encoded by a single-shot spin-echo MRE sequence via a piezo actuator. The waves are transmitted into the mouse skull via a transducer rod. **b** Study design. Serial in vivo MRE imaging of EAE mice starts prior to disease induction (baseline scan, day − 7), followed by EAE induction with PLP_139–151_ and adjuvants (day 0) and imaging and tissue sampling at different time points of the disease course: pre-onset, onset, peak, remission, and relapse. **c** Wave displacement images from one slice of an EAE mouse are shown to illustrate the propagation of shear waves through the brain using frequencies of 1000, 1100, 1200, 1300, and 1400 Hz (thru-plane component). **d** Representative group-averaged stiffness maps (SWS, viridis colormap, *n* = 15 baseline, onset, peak; *n* = 14 pre-onset; *n* = 12 remission; *n* = 7 relapse) at different disease phases

MRI and MRE were performed prior to immunization (baseline) and at the subsequent EAE time points: pre-onset, onset, peak, remission, and relapse. Mice scanned at least 4 times from the longitudinal and cross-sectional groups were included in the analysis of brain stiffness and fluidity (*n* = 7–15). Eight mice of the naïve group were examined by MRE (Fig. 1).

MRI and MRE acquisitions were performed using a 20-mm diameter transmit/receive quadrature volume coil (RAPID Biomedical, Germany) in a 7-Tesla preclinical MRI scanner (BioSpec, Bruker, Germany) with ParaVision 6.0.1 software [63]. A custom-built animal holder delivered a constant airflow with 1.5–2.0% isoflurane in 30% O_2_ and 70% N_2_O via an anesthesia mask. Respiration was constantly monitored with a pressure-sensitive pad placed on the chest (Small Animal Instruments Inc., USA). Body temperature was monitored using a rectal probe and kept stable with integrated warming pads.

An anatomical overview for reference was obtained with a coronal T2-weighted 2D-RARE sequence with the following parameters: repetition time (TR) = 3500 ms, effective echo time (TE) = 33 ms, echo spacing (ΔTE) = 11 ms, RARE factor = 8, 4 averages, 32 contiguous slices of 0.5 mm thickness, field of view (FOV) = 18 mm × 18 mm, matrix size (MTX) = 180 × 180, resolution = 0.1 mm × 0.1 mm × 0.5 mm, bandwidth = 34,722 Hz, and total acquisition time (TA) = 5:08 min.

Quantitative T2-maps were obtained with a coronal standard 2D multi-slice multi-echo (MSME) sequence with the following parameters: TR = 3000 ms, TE = 6.80 ms with 20 echo images, 15 slices of 0.8 mm thickness, FOV = 18 mm × 18 mm, MTX = 180 × 180, resolution = 0.18 × 0.18 × 0.8 mm, and TA = 5:00 min.

Diffusion-weighted imaging (DWI) was performed with the following parameters: 7 slices with a slice thickness of 0.8 mm using a resolution of 0.18 mm × 0.18 mm × 0.8 mm, three *b*-values of 0, 400, 1000 s/mm^2^ in three orthogonal diffusion directions, FOV = 16.2 × 14.4, TE = 21 ms, TR = 3000 ms, and TA = 4:12 min.

MRE was performed using a custom-designed driver system based on a piezoceramic actuator. Harmonic vibrations at 5 frequencies (1000, 1100, 1200, 1300, and 1400 Hz) were consecutively transmitted into the mouse brain via a transmission rod integrated into a customized animal holder (Fig. 1) and sampled using a single-shot spin-echo MRE sequence [63]. Seven coronal slices were acquired with the following imaging parameters: TR = 4000 ms, TE = 53 ms, MTX = 90 × 60, in-plane resolution of 0.18 mm × 0.18 mm, slice thickness of 0.8 mm, FOV = 16.2 × 10.8 mm, and TA = 9 min.

### In vivo MRI and MRE in humans

MRI and MRE were performed in a 3-Tesla MRI scanner (Siemens Lumina, Germany) equipped with a 32-channel head coil. The auto align function was used to position all slices in the center of the brain using the localizer scan. T1-weighted, high-resolution, whole brain images were acquired using a magnetization-prepared rapid acquisition gradient echo sequence (MPRAGE; TE: 2.27 ms, TR: 2300 ms, inversion time: 900 ms, flip angle of 8°, isotropic voxel size of 1 mm^3^). 3D multifrequency MRE with parallel imaging was performed as previously described (GRAPPA [21], acceleration factor 2) using harmonic vibrations generated by pressurized air drivers at 4 frequencies: 20, 25, 30, and 35 Hz. The driver consisted of two plastic bottles placed underneath the subject’s head, which were inflated by compressed air pulses in an alternating fashion. 40 axial slices were acquired using the following imaging parameters: TR = 4700 ms, TE = 70 ms, voxel size = 1.6 × 1.6 × 2 mm^3^, FOV = 202 × 202 mm^2^, and TA = 8 min. In addition, two images with inverted phase-encoding direction were acquired for automated correction of susceptibility-induced distortions [16].

### Tomoelastography parameter reconstruction

Reconstructed wave images acquired at 5 mechanical vibration frequencies (Fig. 1c) were used to derive compound maps of shear wave speed (SWS in m/s) and loss angle of complex shear modulus φ (in rad), which provided surrogate markers of brain stiffness and fluidity, respectively [55]. SWS (in m/s) maps were generated using wave number-based multifrequency dual elasto-visco (k-MDEV) inversion [69] with recently introduced, brain-adapted preprocessing for mice [4] and humans [24, 26], while loss angle (φ in rad) maps were generated using the MDEV inversion method [28]. Mouse brain data were smoothed using a low-pass Gauss filter with a standard deviation of 0.15 mm. Motion- and distortion-corrected wave images were filtered by a 2D low-pass Butterworth filter of first order with a threshold of 600 1/m, followed by spatial filtering of 8 in-plane propagation directions prior to computation of the phase gradient for reconstruction of wave numbers. Human brain data were processed according to Herthum et al*.* [25]. In brief, raw MRI data were motion- and distortion-corrected using SPM12 [52]. Harmonic wave images were corrected for interslice phase discontinuities and smoothed with a low-pass Butterworth filter of first order with a threshold of 200 1/m. Wave images were high-pass filtered using the 3D curl operator, followed by spatial filtering of 20 propagation directions and computation of the 3D phase gradient for reconstructing SWS maps.

### Postprocessing of MRE, T2-maps, and DWI mouse data

MRI and MRE image registration was performed using ANTx2, a custom MATLAB toolbox (latest version available at https://github.com/ChariteExpMri/antx2) [34, 63]. First, MRE magnitude images were 3D-coregistered (affine) and coregistered in a 2D-slice-wise fashion (nonlinear b-spline transformation) to the T2-weighted RARE images using ELASTIX (http://elastix.isi.uu.nl/) [33, 63]. The same spatial transformation was then applied to MRE parameter maps. Second, T2-weighted RARE images were transferred into the Allen mouse brain atlas space (Allen Institute for Brain Science, USA) as described previously [34], and the spatial transformation was applied to all MRE images generated during coregistration in the first step. Finally, MRE values were calculated from the registered MRE parameter maps in the Allen brain atlas space.

T2-maps and DWI image registration was performed by coregistration of the respective images to the T2-weighted 2D-RARE images and transferred to the Allen mouse brain atlas space.

### Generation of segmented brain masks, gray matter probability maps, and disease paradigm

For analysis of regional viscoelastic properties, 9 masks of selected brain regions, as defined by the Allen brain atlas (Allen Reference Atlas – Mouse Brain [brain atlas]. Available from atlas.brain-map.org), were generated using the ANTx2 program: isocortex (here referred to as cortex), hippocampus, caudoputamen, pallidum, thalamus, hypothalamus, midbrain, striatum, and whole brain. The slice range was adjusted for each mask to fit the range of MRE maps. For each animal and each time point, tissue probability masks for gray matter were generated in ANTx2 [1]. Voxels with a probability > 80% were selected to create a gray matter mask, which in the cortex corresponded to about 87% of the original atlas mask. Deep gray matter mask was generated by exclusion of the cortex from the gray matter mask. Due to ventricle enlargement, image registration was erroneous close to the lateral ventricles, therefore individual ventricle masks manually drawn on registered MRE magnitude maps were excluded from the other brain masks, as mechanical wave propagation is not ensured in liquid-filled spaces and cannot be reliably analyzed. Resulting masks were overlaid on MRE parameter maps (SWS and φ-maps), T2-maps (to obtain T2-relaxation times in mm/s), and DWI (to obtain apparent diffusion coefficient (ADC) in mm^2^/s) images, from which mean values were calculated. A relapsing–remitting paradigm predicting continuous tissue softening from baseline to peak, recovery at remission, and another decrease in tissue stiffness at relapse was applied to the averaged stiffness maps, and *P* values of paradigm fit were computed. Data were processed in MATLAB 2020a (Mathworks Inc., USA).

### Postprocessing of human MRE data

Matrices normalized to Montreal Neurosciences Institute (MNI) space were generated from mean MRE magnitude images and applied to SWS maps. SWS maps were normalized to the MNI space using the mean MRE magnitude images to generate averaged parameter maps and tissue probability maps [52]. Probability maps for global brain tissue (whole brain—WB) and gray matter were thresholded at 0.5 to generate segmentation masks. Masks for cortical matter and deep gray matter including nucleus accumbens, nucleus caudatus, globus pallidus, putamen, and thalamus were defined using MNI-labeled atlas regions. Probabilities for cerebrospinal fluid were thresholded at 0.3 and excluded from other masks to avoid tissue–fluid boundary artifacts. Spatially averaged SWS values for MS patients and healthy controls were determined in the following regions: whole brain, cortex, and deep gray matter. All data were processed in MATLAB 2020a (Mathworks Inc. Natick, MN, USA).

### Tissue processing and histology

After imaging, EAE and naive mice were immediately killed with an overdose of ketamine/xylazine and transcardially perfused with 4% paraformaldehyde (PFA) (Carl Roth®, Germany). The brains were fixed with PFA for 24 h at 4 °C. The tissue was dehydrated in 30% sucrose in phosphate-buffered saline (PBS) overnight and then embedded in OCT freezing compound. Coronal sections of 5 µm thickness were cut with a cryostat and kept at − 20 °C until use.

Staining of PNNs was performed in 30 µm-thick brain slices, which were subsequently stored in cryoprotectant solution (25% glycerol, 25% ethylene glycol, 50% 0.1 M PBS) at − 20 °C until use. *Wisteria floribunda* agglutinin (WFA) coupled to FITC (Thermo Scientific, USA) was used for visualization of cortical PNNs [64]. In brief, free-floating sections were washed with PBS, then blocked with 3% goat serum, incubated with WFA FITC (1:1000) overnight at 4 °C, washed 3 times with PBS, stained with 4′,6-diamidino-2-phenylindole (DAPI), and mounted onto glass slides with ImmuMount (Thermo Scientific, USA). For hematoxylin and eosin (HE) staining, 5 µm-thick sections were air-dried, fixed with PFA for 10 min, then washed three times with PBS, and stained with hematoxylin (Roth, Germany) and eosin (Sigma-Aldrich, Germany). For Iba1 staining (1:200; Wako, Fujifilm, Japan), 5 µm-thick sections were air-dried, fixed with PFA for 10 min, then washed three times with PBS, blocked, and incubated with primary antibody overnight at 4 °C. Thereafter, secondary antibody (anti-rabbit, 1:500) was incubated for 1 h followed by PBS washes, DAPI and mounted as for PNNs.

### Microscopic image acquisition and analysis

Images were acquired on an All-in-One Fluorescence microscope BZ-X800 (Keyence, Japan). For visualization of PNNs, z-stacks of the sections were imaged with 20 × magnification, generating TIFF files containing all focal planes. For quantification of WFA^+^ PNNs, images were transformed into 32-bit files, and a universal threshold was applied, using FIJI ImageJ2 [59]. Next, the macro-plugin “Perineuronal net Intensity Program for the Standardization and Quantification of ECM Analysis” (PIPSQUEAK, FIJI version 2.1.0/1.53f) [64] was used for automated PNN intensity analysis, and regions of interest (ROIs) were adjusted manually using the thresholded images. For each mouse, three cortical ROIs of 430 × 430 µm in the left hemisphere and three on the right, when both were available, of one section were analyzed. HE images were captured with 10 × magnification, and the total number of cortical lesions was counted across two different sections per mouse and summed.

### Multiplex immunofluorescence and quantification

Cryosections were fixed in formalin for 1 h at room temperature and dehydrated in an ascending ethanol series. A multiplex was stained by blocking endogenous peroxidase with hydrogen peroxide prior to incubation with anti-iba1 (polyclonal Ab #019-19741, Wako, Fijufilm, Japan), followed by incubation with the EnVision + polymer (Agilent, USA) and the OPAL system according to manufacturer’s instructions (Akoya Biosciences, USA). This staining cycle was repeated with anti-F4/80 (clone D2S9R, Life Technologies, USA). The following OPAL dyes were used for visualization: OPAL-520 for iba1 and OPAL-620 for F4/80. A second multiplex was stained in a similar manner as described above with anti-GFAP (clone 2.2B10, Invitrogen, USA) and visualization with OPAL-540, followed by anti-iba1 and visualization with OPAL-520. Nuclei were stained using 4′,6-diamidine-2′-phenylindole dihydrochloride (DAPI; Sigma-Aldrich, USA), and slides were coverslipped in Fluoromount G (Southern Biotech). Multispectral images were acquired using a Vectra^®^ 3 imaging system (Akoya Biosciences, USA). The inForm software (version 2.4.8) was used for spectral unmixing and cell segmentation as well as cell phenotyping. The cell phenotypes were quantified using R and RStudio (version 1.3.1073).

### Statistics

Statistical analysis was performed using GraphPad Prism 9.0 (GraphPad software, United States) and R version 4.0.2 (R-Foundation, Austria). Normal distribution was tested using the D'Agostino and Pearson test. Repeated measures one-way ANOVA was applied to mean values of SWS and loss angle obtained from the MRE parameter maps averaged over the masks. WFA mean intensity values and the percentage of Iba1^+^ cells were analyzed using one-way ANOVA. Paired *t* tests were performed to compare MRE parameters in the cortex and the remaining gray matter between baseline and peak. A linear mixed model using the lmer function of R [36] was applied for correlation of disease scores with SWS values [36], with the following parameters: outcome = SWS (m/s), independent variable = score, and random effect = animal. The unpaired *t* test followed by *P* value correction for multiple comparisons using the Holm–Bonferroni method was applied to assess differences in SWS between healthy controls and MS patients. Differences were considered statistically significant for *P* values < 0.05.

**Fig. 2:**
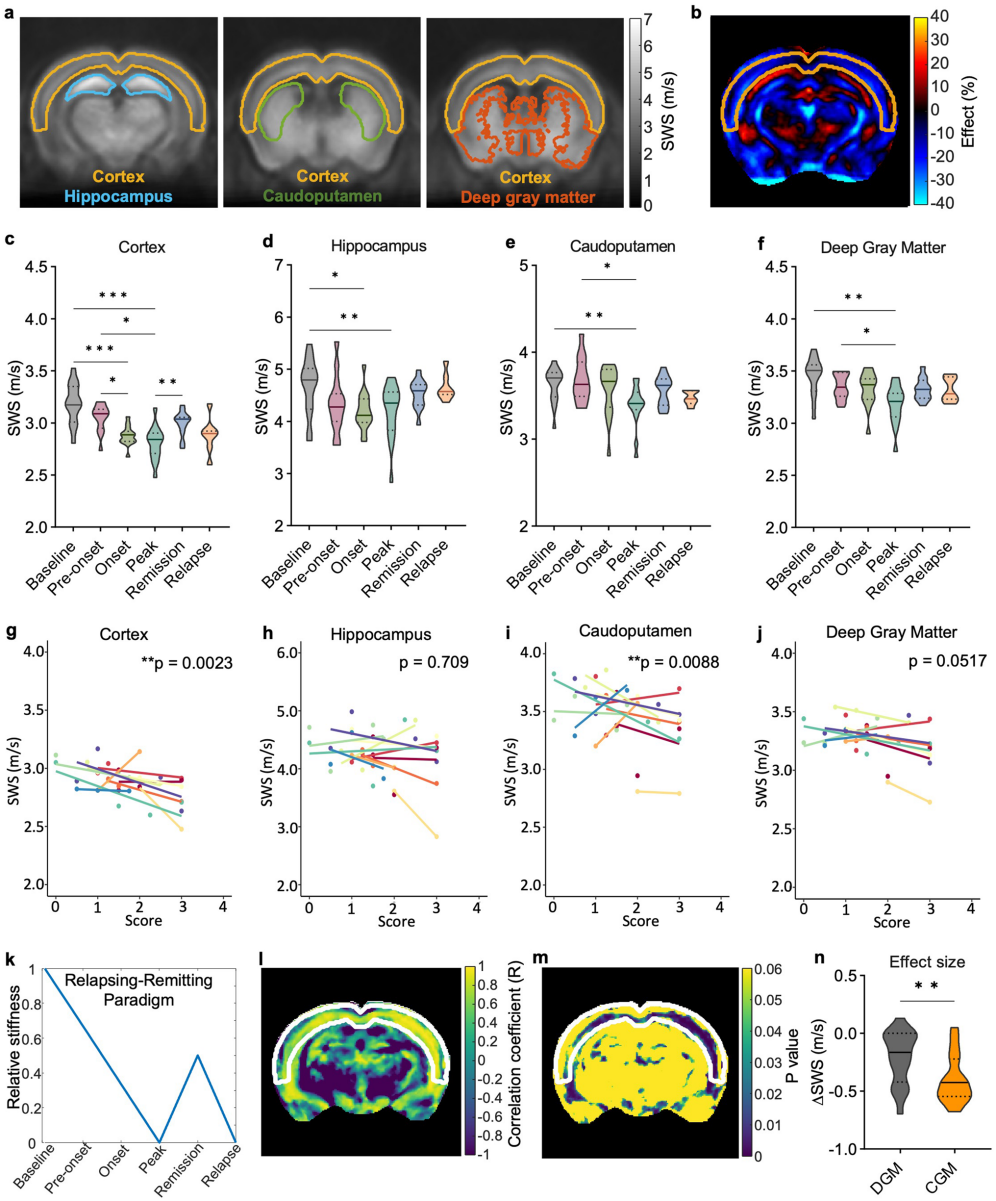
Stiffness measured in the cortical region reflects EAE progression more reliably than in other brain regions. **a** Segmentation of the brain into regions according to the Allen mouse brain atlas overlaid on group-averaged SWS maps (*n* = 15). **b** Effect map of percentage changes in SWS between peak and baseline shows softening throughout the brain including gray matter regions. **c**–**f** Temporal changes in brain stiffness (SWS) in regions significantly affected during EAE (for detailed statistics see Supplementary Table 1);* n* = 15 baseline, onset, peak; *n* = 14 pre-onset; *n* = 12 remission; *n* = 7 relapse. **g**–**j** Disability scores inversely correlate with regional stiffness in the cortex and caudoputamen, but not in hippocampus or deep gray matter; (*n* = 15). **k** A relapsing–remitting paradigm (left-hand side) used for pixel-wise correlation analysis identifies cortical tissue as the best fitting area for assessment of disease progression, as indicated by a high correlation coefficient (**l**) and low *p* values (**m**). **n** Compared with deep gray matter regions, the change in stiffness (ΔSWS) from baseline to peak is significantly greater in the cortex (*n* = 15). *P < 0.05, **P < 0.01, **P < 0.001, ****P < 0.0001

### Study approvals

In vivo animal experiments were conducted in accordance with national and international guidelines for laboratory animal welfare, including directive 2010/63/EU of the European Parliament and of the Council of 22 September 2010, and were approved by the Berlin State Office for Health and Social Affairs (LAGeSo, registration number G0106/19).

Human MRE in volunteers and patients with MS was approved by the ethics committee of Charité-Universitätsmedizin Berlin in accordance with the Ethical Principles for Medical Research Involving Human Subjects of the World Medical Association Declaration of Helsinki (EA1/085/17). Prior to imaging, informed written consent was obtained from all study participants.

## Results

### EAE progression correlates with brain tissue softening measured by MRE

We used serial in vivo multifrequency MRE for longitudinally studying the mouse EAE model in order to determine the mechanical properties of the brain tissue over time and to thus assess cortical pathology during different phases of disease (Fig. 1). Therefore, mice were examined by MRE at six time points (baseline, pre-onset, onset, peak, remission, and relapse) as detailed in the Methods section and outlined in Fig. 1b. From the reconstructed wave images (Fig. 1c), maps of shear wave speed (SWS; in m/s) as a surrogate of brain stiffness [55] were generate as shown on Fig. 1d.

Using a whole brain mask, we confirmed previous reports [44, 54, 73] on tissue softening in the course of EAE (Supplementary Fig. 2). Following EAE induction, all animals showed signs of disease based on the score of ascending paralysis, as shown in Supplementary Fig. 2a. Disease onset was observed on days 9–10 after induction and reached a peak on days 12–14, followed by the remission phase on days 15–21. Relapse occurred on days 22–24 after induction. In the course of EAE, the mice showed a transient decrease in SWS throughout the brain, corresponding to the disease state, with softening at the peak of disease and recovery at remission (Supplementary Fig. 2c; for detailed statistics see Supplementary Table 1). In addition, SWS of the whole brain correlated with disease severity at onset, peak, remission, and relapse (fixed effects estimate = − 0.0598, *P* = 0.0245; Supplementary Fig. 2d). Data on brain fluidity (φ) over the course of EAE are provided in Supplementary Table 2 and Supplementary Fig. 3.

### EAE disability stages are defined by stiffness patterns of the brain cortex

To determine the contribution of cortical stiffness to the global brain softening reported above, we investigated patterns of cortical tissue stiffness over time and compared them with 8 other brain regions (hippocampus, caudoputamen, pallidum, thalamus, hypothalamus, midbrain, striatum, and whole brain) by overlaying the corresponding anatomical masks on atlas-registered SWS maps. Of all regions analyzed, only the cortex, hippocampus, and caudoputamen were significantly affected during EAE (Fig. 2a–f). It is noteworthy that neuroinflammatory alterations mostly involved gray matter areas as demonstrated by the effect map in Fig. 2b. Here, the percentage change from peak to baseline shows 20–30% softening in the cortical regions (yellow mask) (Fig. 2b). Corresponding SWS values for all brain areas and detailed statistics can be found in Supplementary Table 1, while regionally resolved loss angle φ data are provided in Supplementary Table 2.

In the cortex, a significant transient reduction of SWS was observed during onset and peak, but not during remission, with effect sizes that reflect the course of the disease (Fig. 2c and Supplementary Table 1). Similar to findings in the whole brain, the level of disability of the EAE mice correlated with SWS in the cortex (fixed effects estimate = − 0.0737, *P* = 0.0023, Fig. 2g). Of the other brain regions investigated, only the hippocampus showed a significant reduction in SWS at both the peak and onset of disease, while the caudoputamen was affected only at peak disease. However, stiffness changes in these regions did not reflect clinical recovery (Fig. 2d, e; for detailed statistics, see Supplementary Table 1). In the caudoputamen, SWS predicted the disease score (fixed effects estimate = − 0.0965, *P* = 0.0088, Fig. 2i), while in the hippocampus, no effect of the score on SWS was found (fixed effects estimate = − 0.0580, *P* = 0.709) (Fig. 2h).

To confirm the predominant contribution of cortical stiffness to the observed overall softening of gray matter, we compared SWS values of deep gray matter with those of cortical gray matter. Deep gray matter masks revealed significant softening due to neuroinflammation only at peak disease (Fig. 2a, f and Supplementary Table 1). Moreover, as shown in Fig. 2j, signs of disability did not correlate with SWS in deep gray matter regions (fixed effects estimate = − 0.05971, *P* = 0.0517). Furthermore, we observed that the decrease in SWS from baseline to peak was significantly greater in the cortex than in the remaining gray matter regions (*P* = 0.0042, Fig. 2n). Reversible softening of the cortex was further validated as the best predictor of disease progression on assumption that SWS follows the pattern of a relapse-remitting EAE course as shown in Fig. 2k–m. This pattern was derived for the course of stiffness in the brains of EAE mice from the data shown in Fig. 2c–f. The model distinguishes three phases: a linear decrease in stiffness, a short phase of incomplete remission (partial re-stiffening), and final softening during relapse. Overall, our findings indicate that the susceptibility of tissue stiffness to reversible neuroinflammatory processes is specific to the cortical region.

### T2-relaxation times and apparent diffusion coefficient do not distinguish cortical pathology

**Fig. 3:**
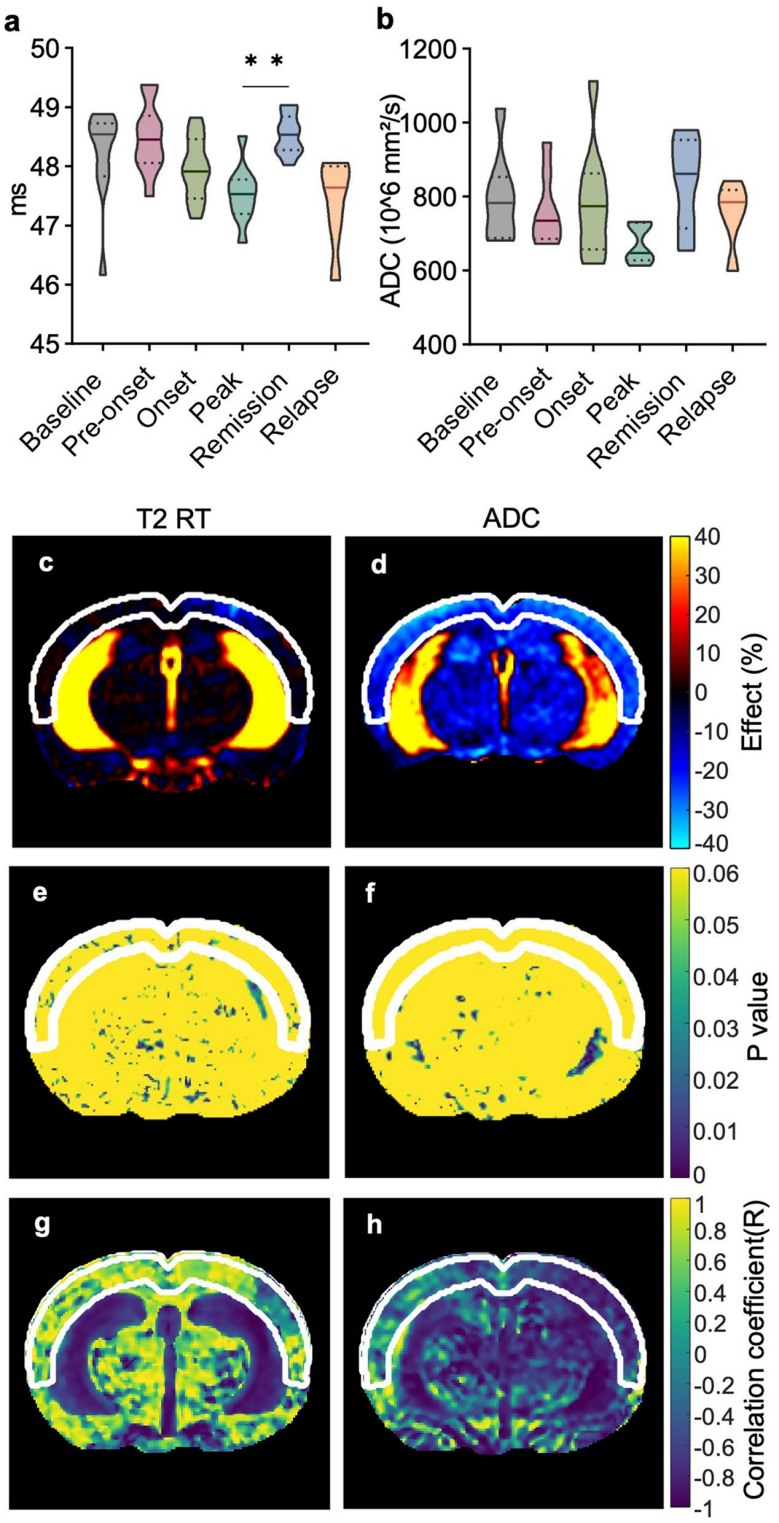
Detection of disseminated water biophysical properties by quantitative MRI. **a** Longitudinal variations in cortical T2 RT in ms (*n* = 9 onset; *n* = 8 baseline, pre-onset; *n* = 7 peak; *n* = 6 remission *n* = 5 relapse); **b** ADC in × 10^6^ mm^2^/s (*n* = 12 baseline, pre-onset, peak; *n* = 11 remission; *n* = 6 relapse). **P < 0.01. **c**, **d** Effect maps of percentage changes in T2 RT and ADC from peak to baseline reveal a focal reduction in T2 RT and a disseminated reduction in ADC in the cortex. **e**–**h** A relapsing–remitting paradigm applied to T2 RT and ADC maps demonstrates a lack of specificity for cortical alterations

To explore possible contributions of other biophysical mechanisms to cortical softening, we investigated whether cortical T2-relaxation time (RT), indicative of free water content, and apparent diffusion coefficient (ADC), associated with water mobility, might reflect and predict the course of EAE. The full range of brain slices for T2 RT and ADC is shown in Supplementary Fig. 1. With both imaging modalities, we observed a pattern similar to that of softening. Reductions of T2 RT and ADC were, however, insufficient to detect inflammation during the peak phase compared with baseline as shown in Fig. 3a, b. Interestingly, both T2 RT and ADC reflected a complete recovery during the remission phase, with a significant increase in RT compared to peak EAE (peak: 47.54 ± 0.48 ms; remission: 48.53 ± 0.34 ms, *P* = 0.0086), indicating the restoration of water-associated biophysical properties, such as edematous swelling, to the level of healthy tissue. The percentage changes from peak to baseline in the effect maps of T2 RT (Fig. 3c) clearly show that a focal effect in the left hemisphere (light blue) drives the average reduction of cortical T2 RT. Apart from that, a relative homogeneity of T2 RT stability is observed across the brain parenchyma (Fig. 3c). Conversely, the ADC effect maps show a distinct reduction in water mobility at the peak of EAE, aligning with a smaller effect in the remission phase, especially affecting the cerebral cortex (Fig. 3d).

Given the predictive power of cortical MRE, which negatively correlated with disease activity, we also tested whether changes in T2 RT and ADC might explain alterations in stiffness based on the relapsing–remitting paradigm. To our surprise, when considered longitudinally, the isolated effects observed on cortical T2 RT were not predictive of disease activity as a high *P* value fitting the model was homogeneously distributed throughout the brain (Fig. 3e, g). Regarding ADC, a nonsignificant correlation was only partially observed in the cortex (Fig. 3f, h). T2 RT and ADC data obtained in different brain regions of EAE mice are provided in Supplementary Tables 3 and 4 and Supplementary Fig. 4.

### Cortical softening is associated with enhanced microglial density but not with the degree of immune cell infiltration

**Fig. 4:**
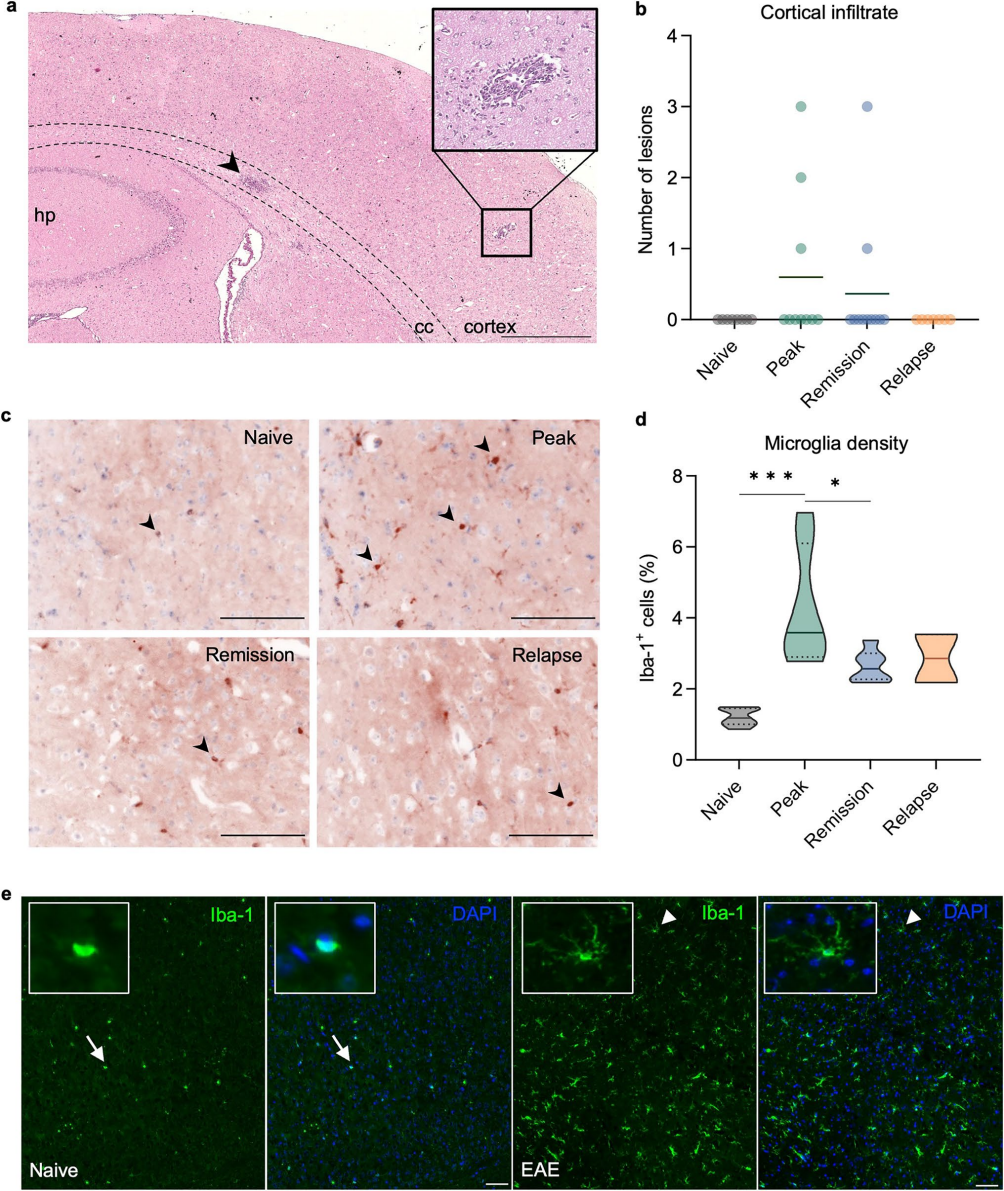
Cortical inflammation occurs independently of leukocyte infiltration and is characterized by microgliosis. **a** HE stain shows lesions in the corpus callosum (arrowhead) and cortical parenchyma (upper panel) (scale bar 500 µm). **b** Quantification of cortical lesions indicates that disease activity is independent of magnitude leukocyte infiltration. **c** Cortical microgliosis is seen at peak EAE, based on higher Iba1^+^ cell counts (arrowheads), compared with a naive brain. **d** Quantification of microgliosis by Iba1+ cell counts; *n* = 7 naive, peak; *n* = 6 remission; *n* = 2 relapse *P < 0.05, ***P < 0.001; scale bar 100 µm. **e** Iba1 immunofluorescence displays distribution and morphological differences between healthy (naïve; white arrow) and EAE (white arrowhead); scale bar 50 µm

A possible impact of leucocyte infiltration on mechanical changes of the cortex was investigated in brain sections stained with hematoxylin and eosin (HE) to detect inflammatory foci. Only 30% of the mice at peak and 18% of the mice in remission showed cortical immune cell infiltrates (Fig. 4a, b), confirming previous studies reporting absence of cortical inflammatory infiltrates during EAE [2, 53]. These results suggest that cortical softening is independent of the number of lesions. However, we found a significant local increase in Iba1^+^ cells, a marker of microglia, within the cortex at peak EAE (naive 0.55 ± 0.46 cells, peak 2.36 ± 1.59 cells, *P* = 0.0001) (Fig. 4c–e). During remission, the percentage of Iba1^+^ cells was significantly reduced compared to peak (Fig. 4d). No changes in F4/80^+^ “resting” microglia were detected in the inflamed cortex. The number of GFAP^+^ astrocytes was increased only during the remission phase, whereas some C3^+^- and connexin43^+^-astrocytes were detected exclusively near the meninges at the peak of EAE (Supplementary Fig. 5).

### PNN remodeling correlates with cortical softening and disease progression

**Fig. 5:**
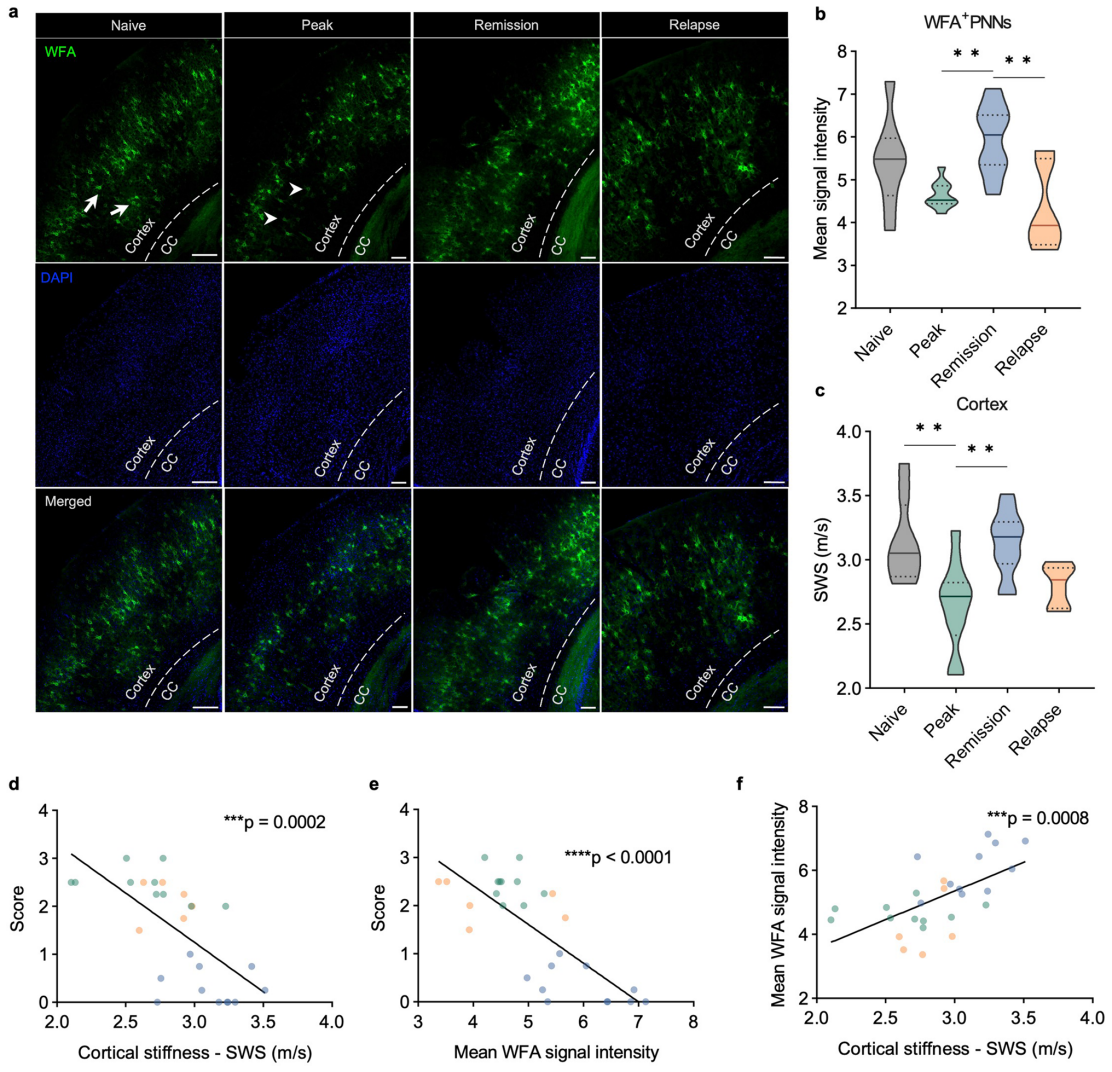
Remodeling of WFA^+^-PNNs in the cortex of EAE mice follows the course of the disease and is correlated with stiffness. **a** Representative immunofluorescence images of WFA^+^-PNNs in the mouse cortex. Remodeling of PNNs is seen as a decrease in green fluorescent signal (baseline: arrows; peak: arrowheads). **b** Quantification of WFA^+^ cortical PNNs in cross-sectional groups in the course of EAE shows a reduction during inflammatory phases, which is partially reversed during remission. **c** Cortical stiffness in cross-sectional groups of mice used for histological analysis shows similar behavior as seen in the longitudinal group; *n* = 8 baseline; *n* = 10 peak; *n* = 11 remission; *n* = 6 relapse. **d** WFA^+^-PNN remodeling correlates with the disease course. **e**, **f** Changes in cortical stiffness correlate with the disease course and WFA signal intensity; *n* = 10 peak; *n* = 11 remission; *n* = 6 relapse *P < 0.05, **P < 0.01, ***P < 0.001

It has been reported that cortical microgliosis may contribute to ECM degradation and PNN remodeling [53]. Therefore, we investigated whether degradation of PNNs, stained with *Wisteria floribunda* agglutinin (WFA), was associated with brain softening in EAE. Averaged WFA signal intensity was quantified across the cortex, including areas comprising the retrosplenial cortex, the somatosensory cortex, and the auditory cortex (Fig. 5a). As EAE progressed, WFA signal intensity decreased, with the lowest intensity at peak disease (naive 5.41 ± 1.1; peak 4.64 ± 0.32), pointing to inflammation-induced remodeling of cortical PNNs in EAE (Fig. 5b). PNN remodeling was reversed during the remission phase (*P* = 0.0041, mean signal intensity of 4.64 ± 0.32 during peak disease vs. 5.94 ± 0.81 during remission) and decreased again during relapse (*P* = 0.0018, 5.94 ± 0.81 during remission vs. 4.3 ± 1.0 during relapse), consistent with the pattern of cortical softening shown in Fig. 2b. In addition, PNN remodeling significantly correlated with the EAE severity score (*P* < 0.0001, *r* = − 0.77) (Fig. 5d). Cortical SWS values for each cross-sectional group are shown in Fig. 5c. Cortical softening correlated significantly with disease severity in the cross-sectional group, confirming the pattern identified in the longitudinal group (*P* = 0.0002, *r* = − 0.66) (Fig. 5e). Strikingly, WFA^+^-PNN signal intensity positively correlated with changes in cortical stiffness in the course of EAE (Fig. 5f).

### Cortical softening is a pathological hallmark of MS

**Fig. 6:**
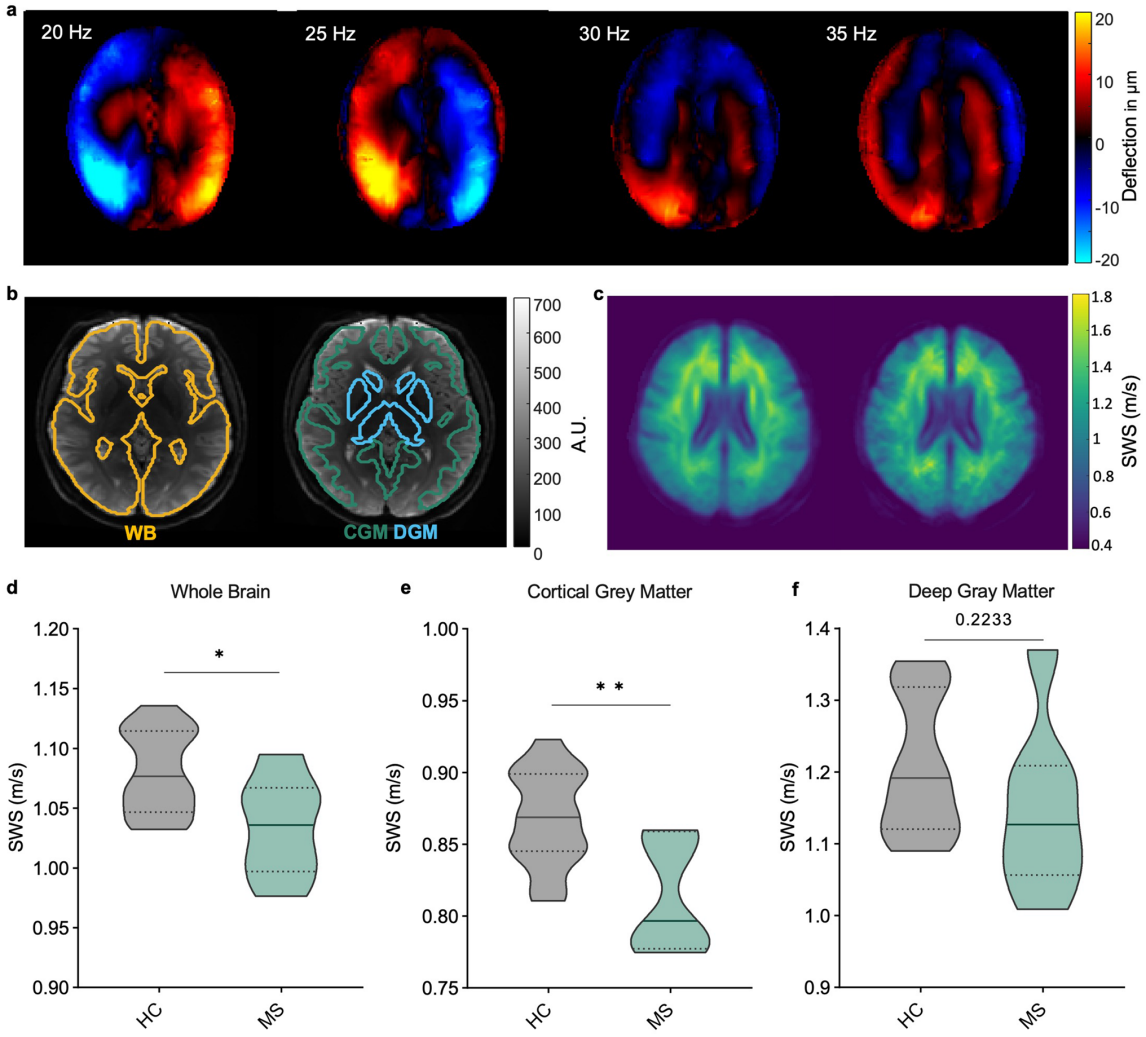
Cortical softening as a signature of MS tissue remodeling. **a** Shear wave propagation through the human brain at 20, 25, 30, and 35 Hz (one central slice, thru-plane wave component). **b** Group-averaged SWS maps (rainbow color scale) of HCs (*n* = 14, left-hand side) and MS patients (*n* = 12, right) show visually apparent disseminated softening throughout the brain and marked effect sizes within cortical gray matter areas in the MS group. **c** Examples of whole brain (WB), cortical gray matter (CGM); and deep gray matter (DGM) masks. Group statistics of MS-related tissue softening in whole brain **d**, cortical gray matter **e**, and deep gray matter **f**; *P < 0.05, **P < 0.01

To evaluate the translational value of cortical softening as a novel imaging marker in MS, we investigated multifrequency MRE with tomoelastography postprocessing in patients (remitting-relapsing MS, *n* = 12, mean age = 38 ± 5 years, mean EDSS score = 2.2 ± 1.32, mean disease duration = 6 ± 5 years) and healthy controls (HC) (*n* = 14, mean age = 38 ± 9 years) (Fig. 6a–c). First, our results confirmed the previously reported global decrease in brain stiffness in MS [67, 75] (MS: 1.05 ± 0.04 m/s; HC: 1.08 ± 0.03 m/s; *P* = 0.0369) (Fig. 6d). Beyond this, novel cerebral tomoelastography allowed us to analyze effects of MS on cortical gray matter (CGM) for the first time. CGM softened significantly more markedly than deep gray matter (DGM) (CGM HC: 0.87 ± 0.03 m/s; CGM MS: 0.82 ± 0.04 m/s, *P* = 0.0036; DGM HC: 1.21 ± 0.09 m/s; DGM MS: 1.14 ± 0.09 m/s, *P* = 0.0594) (Fig. 6e, f). These results coincide with our data obtained in EAE mice and suggest that pathological alterations are specific to the cortical region. Importantly, no differences in stiffness were detectable within the HC group when stratified by age or sex (Supplementary Fig. 6).

## Discussion

In the study presented here, we addressed key aspects of MRE development towards detection and staging of neuroinflammation in MS. First, our results show that cerebral tomoelastography can reliably measure cortical brain softening in both the EAE mouse model and MS patients. In contrast, quantitative MRI based on T2-weighted and ADC imaging did not reflect the relapsing–remitting course of the disease with partial recovery during remission in mice. Second, we demonstrate that the degree of cortical softening correlates with disease severity in mice, suggesting that MRE has the potential for staging inflammatory activity in MS patients. Finally, our results show that cortical ECM remodeling, as reflected by PNN integrity, correlates with cortical softening during neuroinflammation. Since proper neuronal function critically depends on intact PNNs and their surrounding ECM, inflammation-induced PNN degradation may contribute to cognitive impairment in patients with MS as shown by Paylor et al. [50].

Corroborating previous studies [2, 44, 54, 63, 73], our results show that mice with relapsing–remitting EAE have softer brains than control mice. Moreover, we here, for the first time, show that MRE can detect distinct EAE phases and that brain softening correlates with the disability score. Strikingly, we observed a partial recovery of brain stiffness during remission following the marked reduction in stiffness at peak disease, suggesting that MRE can distinguish between inflammatory and recovery phases in the course of MS. Although the conventional EAE scoring system primarily assesses disability related to spinal cord damage, and not to brain alterations it is well established that the relapsing–remitting EAE involves the brain [11] and that the degree of brain alterations parallels the levels of spinal cord damage. Thus, brain affection can be indirectly reflected by the conventional scoring system [2, 44, 54, 62, 63, 73]. It has been shown that in the cortex, as in the spinal cord, meningeal inflammation results in damage to adjacent cortical areas [6]. This suggests that even in the absence of significant leukocyte infiltration, inflammation initiated and even confined to the meninges can spread to the cortical parenchyma and contribute to changes in mechanical properties.

Another key element of our analysis pipeline was image registration and automated atlas-based segmentation. The obtained group maps revealed unique patterns of EAE-associated softening, most markedly in the cortex. The hippocampus and caudoputamen were also affected, but to a lesser extent, while other brain regions remained unchanged. Of the brain regions with altered stiffness, only the hippocampus showed additional changes in loss angle φ, which represents tissue fluidity. The altered fluid properties of hippocampal tissue may be associated with activity of the neurogenic niche of the mouse brain, which was shown to exhibit reduced stiffness and fluidity in vivo [46].

Using quantitative MRI, we gained deeper insights into possible in vivo mechanisms of brain softening in neuroinflammatory conditions. The observed decrease in T2-relaxation times rules out vasogenic edema as a cause of tissue softening. Published data suggest that T2-relaxation time increases with water accumulation in edematous tissue, while it decreases with the release of iron and macromolecules during nonspecific demyelination processes in MS [23]. Thus, the observed decrease in T2 relaxation is consistent with demyelination. Furthermore, the decrease in ADC found in our study suggests a mechanism independent of extracellular water accumulation. Instead, ADC is known to decrease when water molecules are translocated from the ECM into cellular compartments, such as during the formation of cytotoxic edema [5]. However, these mechanisms are not primary targets for disease monitoring in MS, as T2-weighted and ADC-based MRI parameters have limited sensitivity for disease disability in MS. Conversely, MRE has turned out to be specific for cortical pathology, further corroborating the potential of tissue softening as an imaging marker in MS.

In EAE, lower brain stiffness has been consistently reported and was associated with perivascular deposition of ECM [73], presence of inflammatory cells [44, 75], and magnetic particle accumulation as a sign of gliosis and/or ECM remodeling [63]. The observation that cortical softening in EAE is reversible suggests that the observed changes in mechanical properties are governed by transient neuroinflammatory events rather than permanent neurodegenerative processes. Strikingly, stiffness changes in our study were not associated with inflammatory immune cell infiltrates and occurred in areas with only a few perivascular lesions. Thus, clinically relevant alterations of cortical tissue are linked to diffuse inflammation rather than leukocyte infiltration and formation of perivascular lesions. This may explain why MRI markers related to MS lesions are often unspecific and not sensitive to clinical severity and cognitive impairment [17]. Although detection of gray matter lesions by MRI is improved at higher field strength, it is important to acknowledge that even ultra-high-field MRI at 7 T only provides a superficial understanding of cortical demyelination, in comparison to histopathological detection [42] Moreover, the availability of 7 T MRI for multi-center, clinical trials is still limited. Indeed, unlike white matter lesions, most cortical lesions in MS are thought to be noninflammatory, i.e., without leukocyte infiltration or blood–brain barrier disruption [30]. Still, cortical lesions are often accompanied by meningeal inflammation [29, 31, 66], which has been shown to promote microglial activation, proliferation and, ultimately, cortical damage [40, 51, 71]. This is in agreement with our observation of an increased density of Iba1^+^ microglia at peak EAE, followed by its drop during remission. The reversibility of decreased T2-relaxation time in the remission phase also corroborates a decrease in the number of microglia, as a source of iron deposition. Morphological changes of cortical microglia were not quantified except for an increase in microglial density. The implication of microglia morphology in cortical softening should be investigated in depth in future studies. We found no evidence of direct involvement of other cell types on tissue stiffness, such as astrocytes or macrophages, at the peak of cortical inflammation, although some C3^+^ and connexin 43^+^-astrocytes were found near the meninges, possibly exerting an indirect effect via production of soluble factors. It has been shown that, even in the absence of detectable leukocyte infiltration, microglia in the frontal cortex are activated during the first hours of acute EAE, accompanied by the production of soluble factors such as the proinflammatory cytokine TNF-α [10] and the matrix metalloproteinase MMP9 [18]. This led us to investigate PNN integrity. The products of activated microglia such as MMP9 are known to degrade PNNs in perivascular cuffs of MS lesions [19]. WFA^+^-PNNs form a specialized ECM that sheathes soma and dendritic spines and are found mainly in parvalbumin-positive interneurons and some pyramidal cells in the mouse cortex [14]. Indeed, quantification of WFA^+^-PNNs revealed a clear transitory loss of WFA signal consistent with the course of inflammation in EAE mice, which correlated remarkably well with both the disease score and cortical softening.

**Fig. 7:**
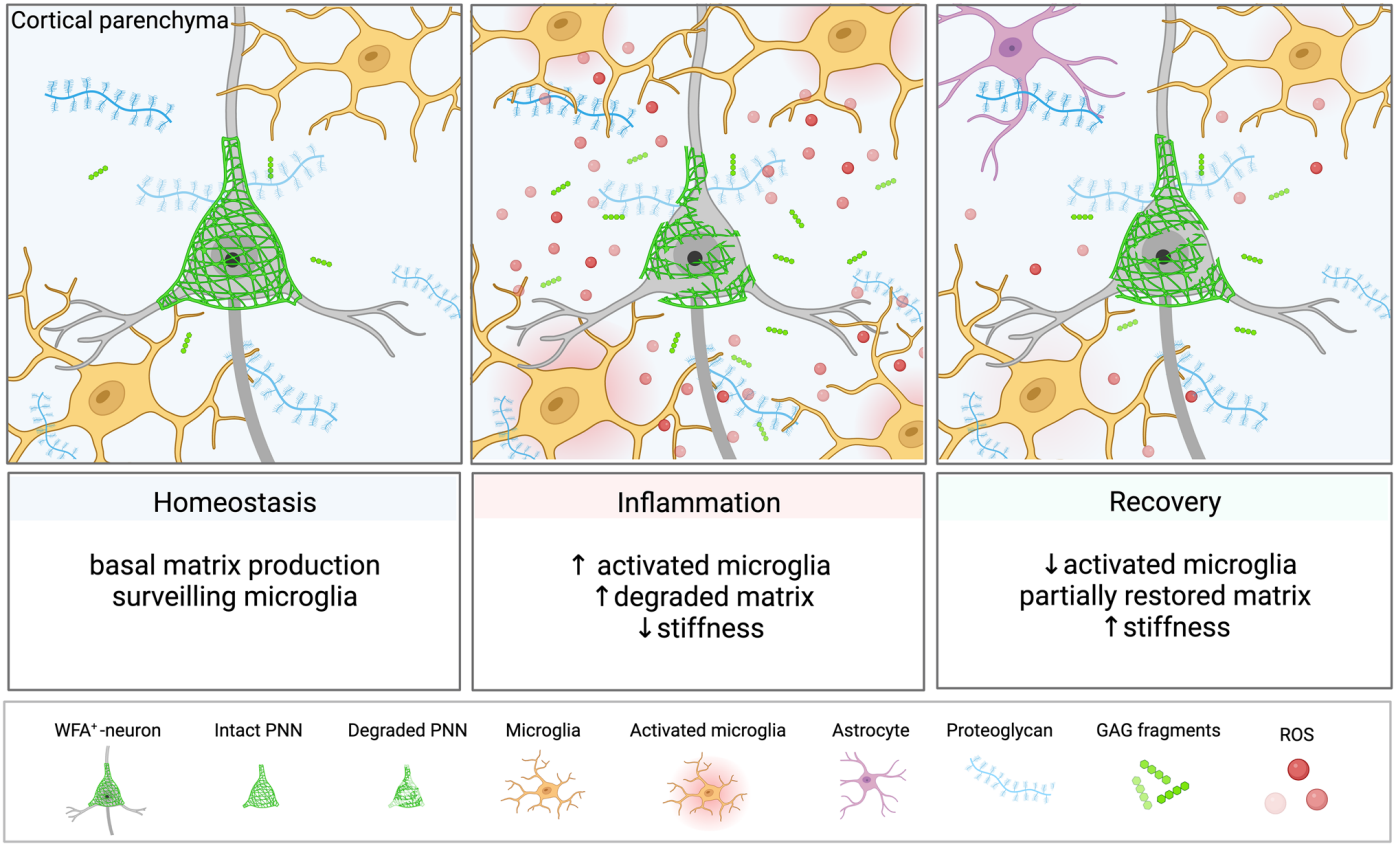
Overview of cortical microstructural remodeling behind transient softening during EAE. In homeostatic conditions, ECM molecules are constantly produced by neurons and glial cells. Cortical microglia are found in low numbers surveilling the tissue. As stiffness transiently drops in the inflammatory phases, a reversible neuroinflammatory process affecting tissue microstructure drives the mechanical changes. The proposed hypothesis involves the activation and proliferation of microglial cells in the cortex via interaction with meninges-derived soluble factors, i.e., cytokines, diffusing through the parenchyma. Activated microglia release matrix-degrading elements, such as proteases and reactive oxygen species (ROS), to induce matrix degradation as reflected by remodeled PNNs. In the recovery phase, lower inflammatory activity leads to a decrease in the number of microglia, allowing restoration of normal matrix and PNN composition. In this phase, the tissue microenvironment does not return to its original state, still displaying stiffness clearly distinct from healthy tissue. Thus, an interplay between PNN remodeling as well as overall matrix alterations and local cellular composition produces the observed transitory cortical softening, which can thus be used as a quantitative marker of neuroinflammation. Created with BioRender.com

WFA binding to PNNs has been suggested to be sensitive to changes in CS profile [58]. Recently, we have demonstrated that EAE-induced inflammation degrades CS glycosaminoglycans and alters the CS sulfation pattern [62]. Although alterations in the cortical CS sulfation pattern remain to be investigated further, structural changes in CS may be responsible for the PNN remodeling detected by changes WFA signal intensity in the course of EAE. Thus, CS degradation may explain the PNN changes observed in our study. PNN remodeling, mediated by microglial activation, has been shown to play a critical role in neuronal plasticity [14, 53] and progressive neurodegeneration in MS [19, 70] and EAE [53]. In line with our findings, Schregel et al. reported lower stiffness in focal cortical lesions produced by focused ultrasound in combination with MOG-induced EAE [60]. These focal lesions, like those in our PLP-induced EAE model, were characterized by activated microglia and resemble pre-active lesions in MS [60]. Consequently, PNN remodeling and loss of PNN integrity as quantified by MRE may be a promising target for detection of clinically relevant tissue alterations in MS (Fig. 7).

In agreement with our observations in mice, the group of MS patients in our study exhibited disseminated global brain softening, as reported previously for primary and secondary progressive MS [67] as well as relapsing–remitting MS [75]. Here, RRMS patients in remission were studied. Unlike EAE remission, which in most cases is associated with complete recovery and a score of 0, MS patients in remission are in most cases not free of inflammation and disease activity. Therefore, MS patients in remission cannot be considered healthy.

Moreover, the novel tomoelastography approached used here allowed us to overcome current limitations of clinical cerebral MRE and to analyze the stiffness of the fine cortical layer in patients quite similar to the stiffness analyses performed in the mouse brain. While tomoelastography of the mouse brain has been used in several recent studies [2, 4, 22, 63], ours is the first study using the corresponding method in MS patients. A major reason why cerebral tomoelastography is more challenging in humans is the presence of numerous slip interfaces in the human brain compared with the relatively smooth mouse brain. Our group has recently published a way to overcome slip interface artifacts in volunteers [43]. As this technique has been shown to be highly reproducible even at 1-year follow-up, it could be used to monitor treatment response in serial examinations without the need for administration of a contrast agent [25].

Although encouraging, our study has limitations. Other structural changes causing brain softening such as cortical demyelination, as reported in MS patients [31], might have played a role. Although there is limited evidence regarding cortical demyelination in mice, we cannot fully rule out that our MRE data were, at least partially, affected by processes associated with de- or remyelination. Moreover, we should include additional pre-onset time points to assess stiffness changes before clinical manifestations of the disease become apparent. While we and others have shown that inflammatory processes occur in the brain before the onset of clinical signs of EAE [45], the exact timing of such early effects remains unclear. Thus, using a time point later than day 5 as a pre-onset time point may have revealed these early inflammatory changes in our experiments. Finally, the inclusion of more relapsing animals would help detecting significant stiffness changes at this timepoint.

In conclusion, using cerebral MRE based on novel tomoelastography in the EAE mouse model, we have shown that cortical stiffness is sensitive to cortical ECM remodeling and can be used as an imaging marker of disease progression in remitting-relapsing MS. Moreover, our results show that transient softening of the brain cortex correlates with remodeling of cortical WFA^+^ PNNs, while it appears to be independent of the extent of local leukocyte infiltration. In the comparison of MS patients with healthy volunteers, cerebral tomoelastography confirms predominant softening of cortical areas, thus corroborating the translational value of our observations made in the EAE model. This opens the perspective of leveraging the soft signature of cortical inflammation for noninvasive and quantitative imaging without the need for contrast agent use in monitoring the treatment response of MS patients.

### Supplementary Information

Below is the link to the electronic supplementary material.

Supplementary file1 (DOCX 3957 KB)

## Data Availability

All data are available in the main text or the supplementary materials.
