# Supplementary material for: Cortical matrix remodeling as a hallmark of relapsing–remitting neuroinflammation in MR elastography and quantitative MRI
Source: Acta Neuropathol. 2024 Jan 4;147(1):8. doi: 10.1007/s00401-023-02658-x (PMC10766667; doi:10.1007/s00401-023-02658-x)
Supplement: Supplementary file 1 — Supplementary file1 (DOCX 3957 KB) [file 401_2023_2658_MOESM1_ESM.docx]

# Supplementary information

**Cortical matrix remodeling as a hallmark of relapsing-remitting neuroinflammation in MR elastography and quantitative MRI**

Rafaela V. Silva^†^; Anna S. Morr^†^; Helge Herthum; Stefan P. Koch; Susanne Mueller; Clara S. Batzdorf; Gergely Bertalan; Tom Meyer; Heiko Tzschätzsch, Anja A. Kühl, Philipp Boehm-Sturm; Jürgen Braun; Michael Scheel; Friedemann Paul; Carmen Infante-Duarte^*^; Ingolf Sack^*^.

^†^These authors contributed equally to this work and share first authorship

^*^These authors contributed equally to this work and share senior authorship

Corresponding author: Ingolf Sack & Carmen Infante-Duarte

[ingolf.sack@charite.de](mailto:ingolf.sack@charite.de) / [carmen.infante@charite.de](mailto:carmen.infante@charite.de)

**This file includes:**

Supplementary Figures 1 to 6 and Supplementary Tables 1 to 4.


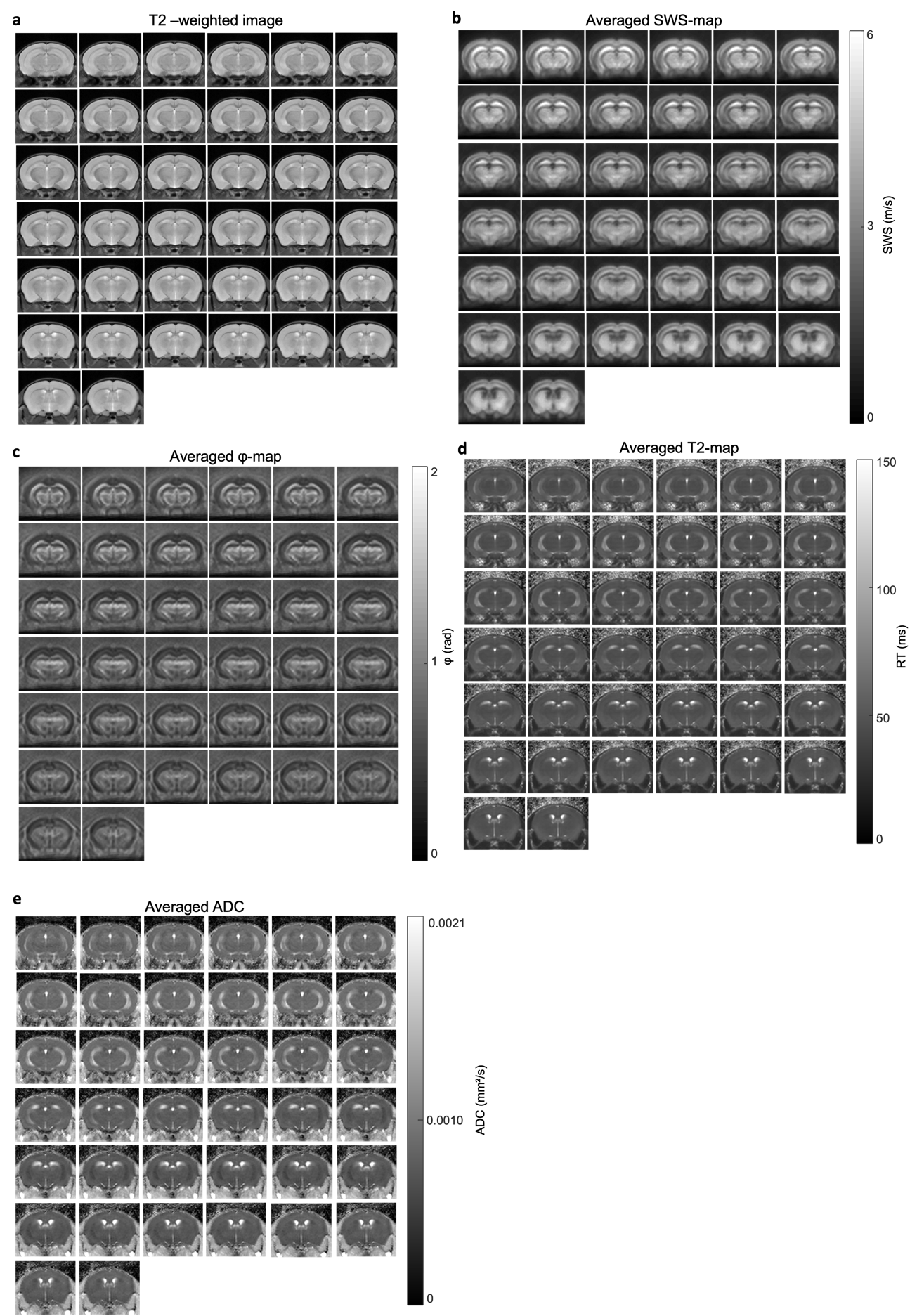


**Supplementary Fig. 1: Full range of brain slices imaged with multifrequency MRE and quantitative MRI.** Brain slices comprising bregma -2.84 mm to -0.25 mm display anatomical features on T2-weighted images (**a**), corresponding resolved stiffness (**b**) and fluidity maps (**c**), T2 relaxation times maps (**d**), and ADC images (**e**) at baseline.


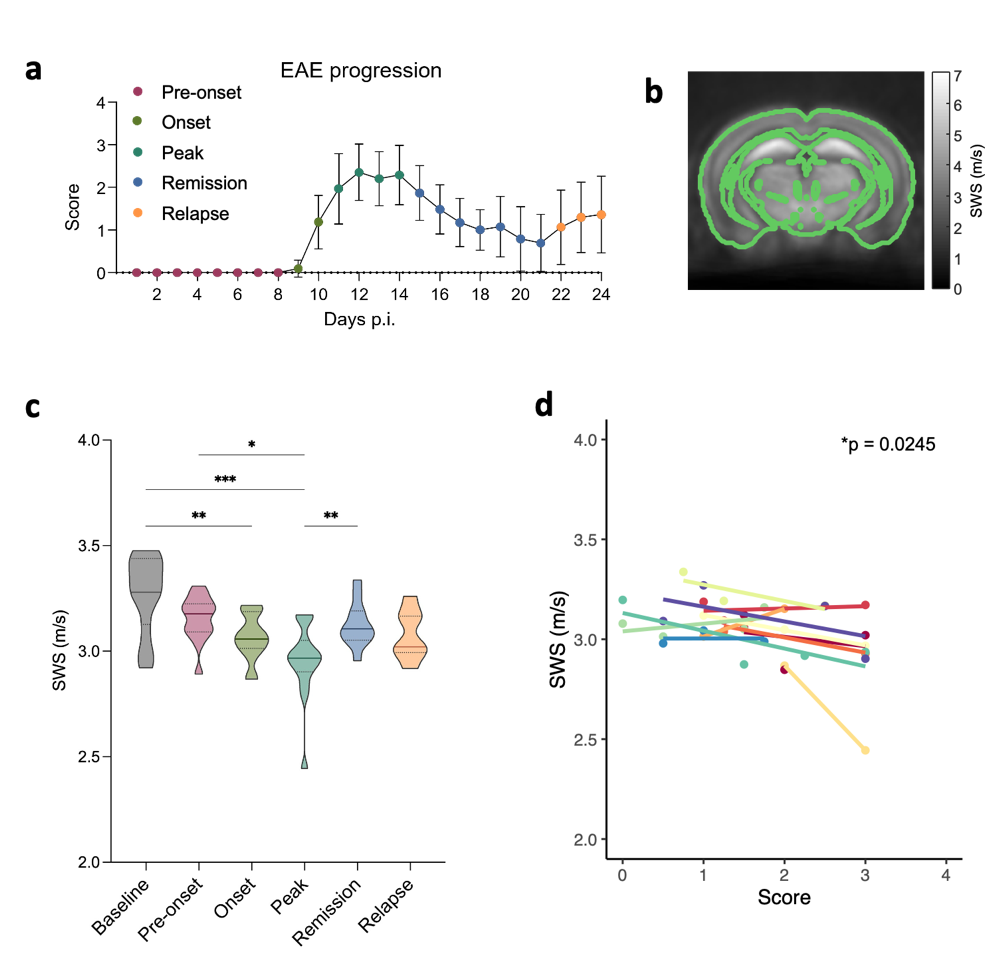


**Supplementary Fig. 2: EAE progression is associated with global brain softening detected by MRE.** **a** Disease progression after immunization with disability scores reflecting the course of relapsing-remitting EAE. **b** Averaged SWS map (grayscale, n = 15) showing the local variation in stiffness across the mouse brain at baseline with a mask for the whole brain according to Allen Mouse Brain atlas (green lines overlaid on grayscale SWS maps). **c** Transient global softening is observed during EAE (for statistics see Table 1) and correlates inversely with disease score (**d**). n = 15 baseline, onset, peak; n = 14 pre-onset; n = 12 remission; n = 7 relapse; *p<0.05, **p<0.01, ***p<0.001.


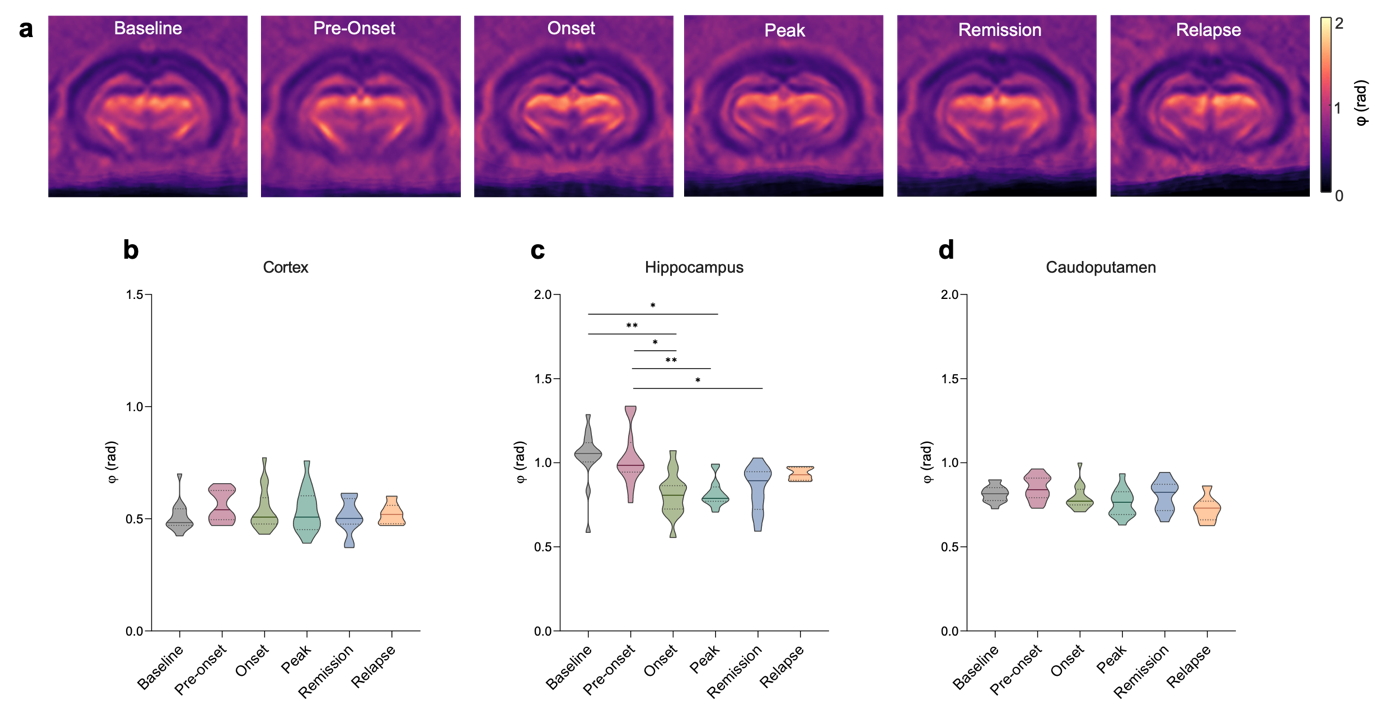


**Supplementary Fig. 3: Brain fluidity quantified by the loss angle 𝝋 is less sensitive to EAE progression than stiffness.** (**a**) Averaged 𝝋 maps (magma colormap, n = 15) showing the local variation at fluidity across the mouse brain at baseline, pre-onset, onset, peak, remission, and relapse. No significant changes in brain tissue fluidity are observed for the whole brain (**b**) or areas with detected changes in stiffness: cortex (**c**), hippocampus (**d**), and caudoputamen (**e**). n = 15 baseline, onset, peak; n = 14 pre-onset; n = 12 remission; n = 7 relapse.


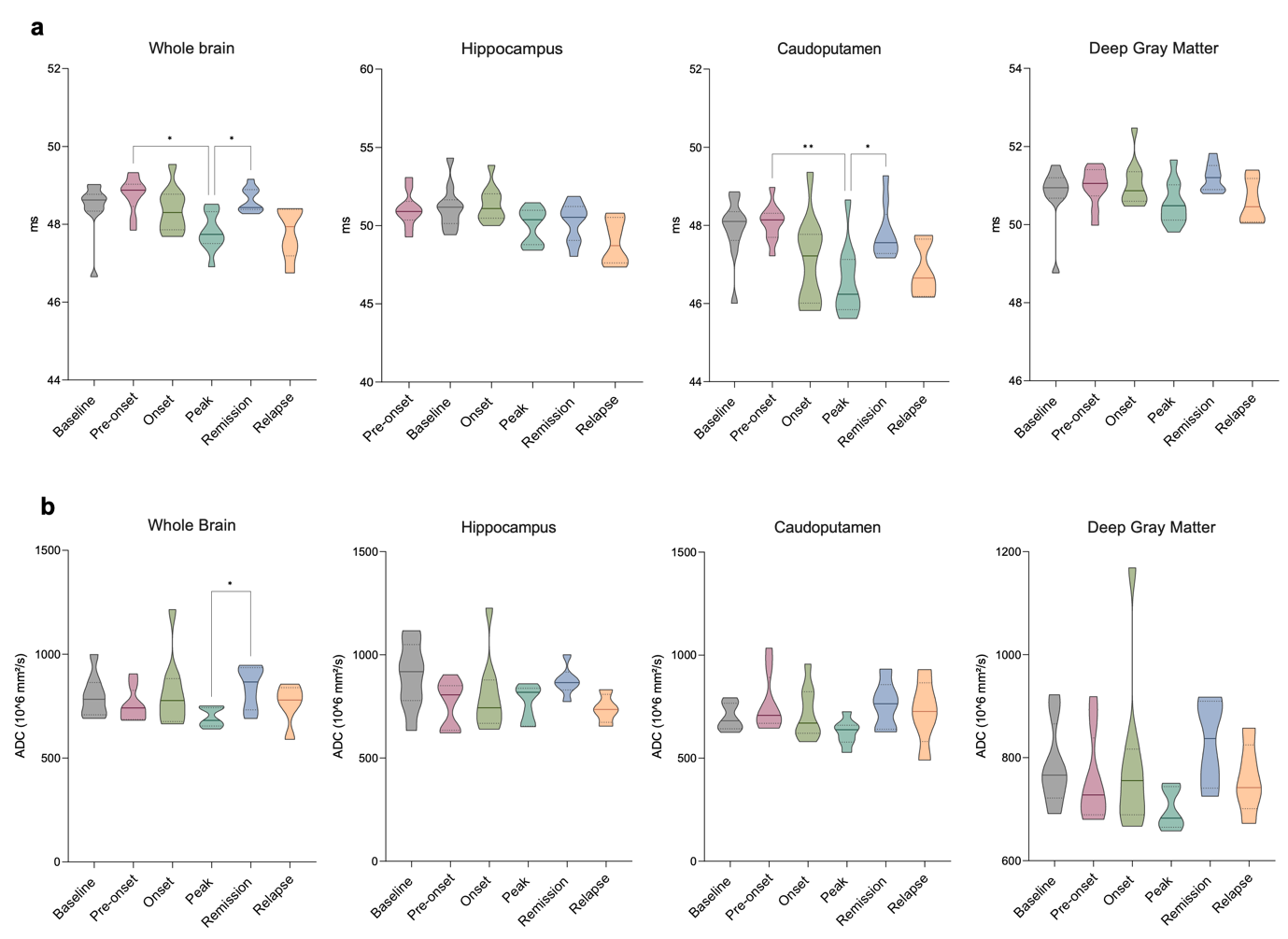


**Supplementary Fig. 4: T2 relaxation times and ADCs over the course of EAE.** **a** T2 relaxation times in the whole brain, hippocampus, caudoputamen, and deep gray matter (n = 9 onset; n = 8 baseline, pre-onset; n = 7 peak; n = 6 remission n = 5 relapse). **b** DWI-derived ADCs in whole brain, hippocampus, caudoputamen, and deep gray matter (n = 12 baseline, pre-onset, peak; n = 11 remission; n = 6 relapse).


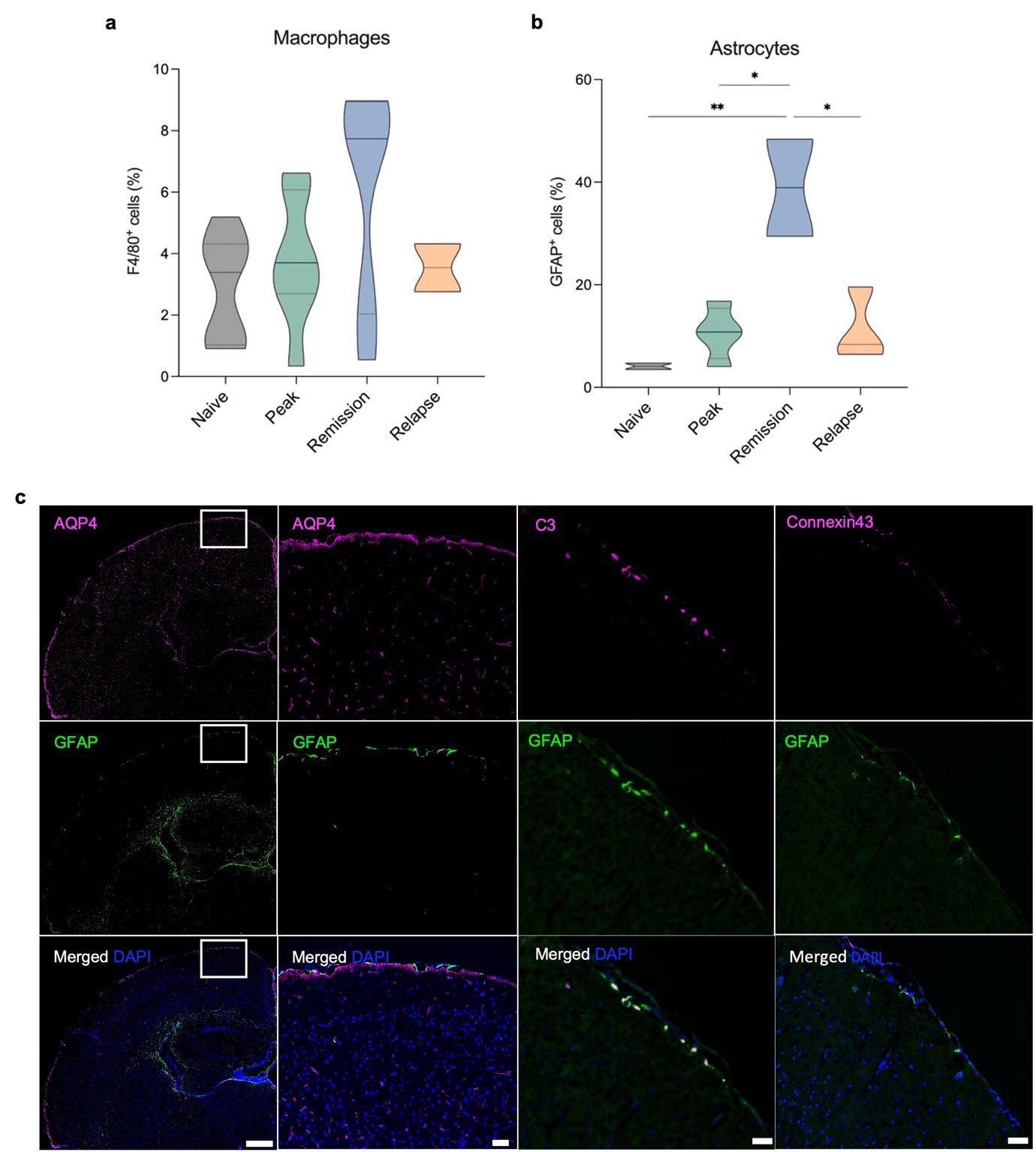


**Supplementary Fig. 5: Quantification of inflammatory cells in the cortex over the course of EAE.** **a** Macrophages (F4/80+ cell counts) did not significantly change during EAE (n = 7 naive, peak; n = 6 remission; n = 2 relapse *p<0.05, **p<0.01). **b** Astrocytes (GFAP+ cell counts, right) were only increased late disease, during the remission phase (n = 2 naive, n = 4 peak; n = 2 remission; n = 3 relapse *p<0.05, **p<0.01). **c** Cortical astrocytes at peak EAE. While AQP4+astrocytic processed are expressed throughout the entire brain and especially prominent at the borders, GFAP+astrocytes are found largely found at the brain borders and around ventricles. Magnified areas show distribution of both markers in the cortex, with absence of GFAP+astrocytes in the deep parenchyma. GFAP+ astrocytes were positive for C3 or connexin 43, as activation markers, only at the border with the meninges, but not in the cortical parenchyma.


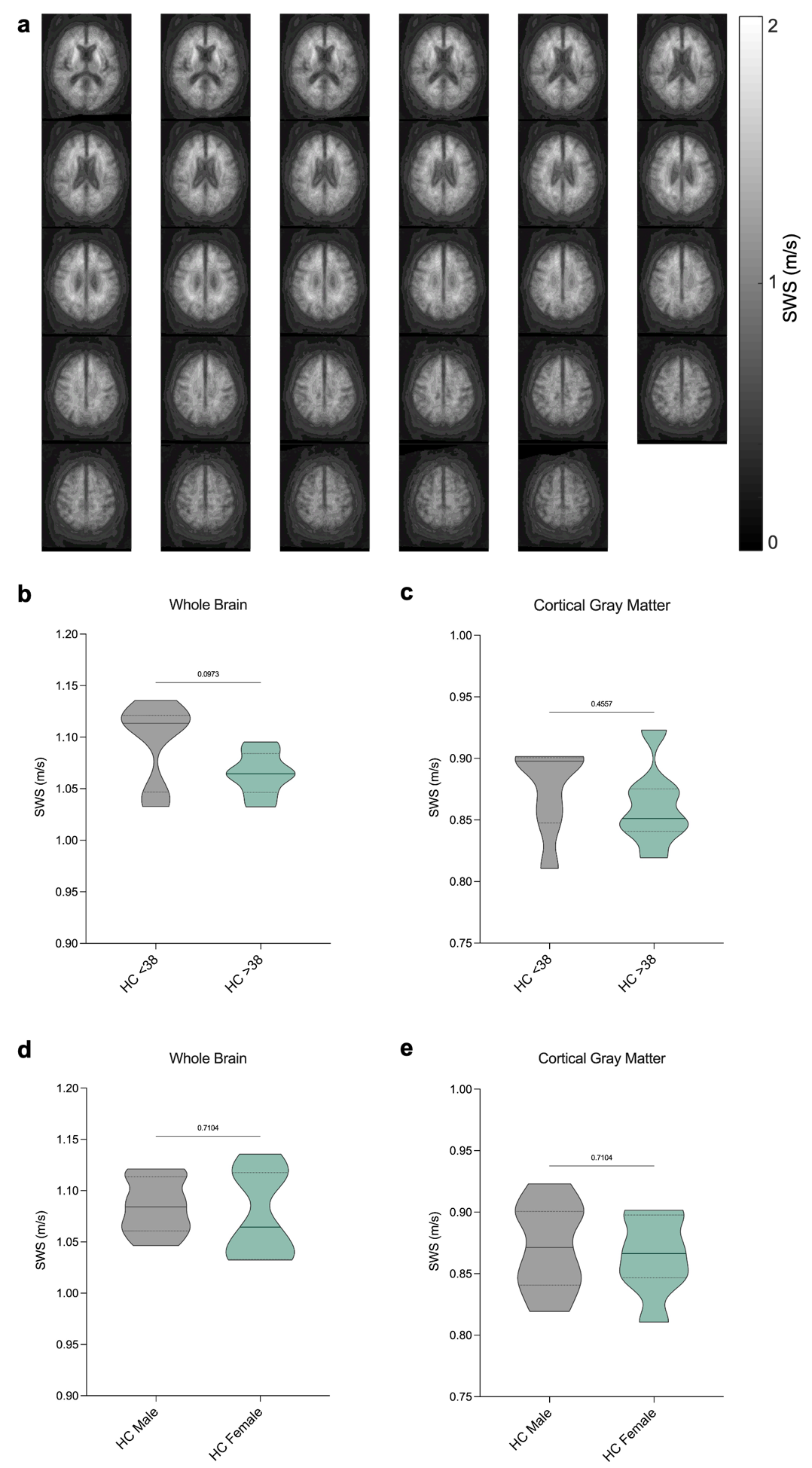


**Supplementary Fig. 6: Range of human brain slices acquired with multifrequency MRE and stiffness changes among healthy volunteers adjusted for age or sex. a** Group averaged stiffness of the human brain slices visualized by MRE. No differences in stiffness were observed when findings in healthy controls were adjusted for age (**b-c**) and for sex (**d-e**).

**Supplementary Table 1.**

Regional stiffness in the mouse brain given in shear wave speed. p < 0.05 is given in bold.

| **Region** | **Timepoint** | **Mean ± SD (m/s)** | **Adjusted *p* value** | | | | |
| --- | --- | --- | --- | --- | --- | --- | --- |
|  |  |  | **Pre-onset** | **Onset** | **Peak** | **Remission** | **Relapse** |
| **Whole Brain** | Baseline | 3.26 ± 0.13 | 0.3724 | **0.0087** | **0.0010** | 0.2487 | 0.1094 |
|  | Pre-onset | 3.15 ± 0.11 |  | 0.3125 | **0.0271** | 0.9839 | 0.6533 |
|  | Onset | 3.06 ± 0.11 |  |  | 0.1159 | 0.6378 | 0.9996 |
|  | Peak | 2.95 ± 0.18 |  |  |  | **0.0031** | 0.1450 |
|  | Remission | 3.12 ± 0.11 |  |  |  |  | 0.9436 |
|  | Relapse | 3.08 ± 0.13 |  |  |  |  |  |
| **Cortex** | Baseline | 3.19 ± 0.14 | 0.2961 | **0.0002** | **0.0002** | 0.1203 | 0.0510 |
|  | Pre-onset | 3.05 ± 0.13 |  | **0.0140** | **0.0120** | 0.8822 | 0.2496 |
|  | Onset | 2.87 ± 0.10 |  |  | 0.5783 | 0.0883 | >0.9999 |
|  | Peak | 2.80 ± 0.17 |  |  |  | **0.0029** | 0.7047 |
|  | Remission | 2.99 ± 0.12 |  |  |  |  | 0.6578 |
|  | Relapse | 2.88 ± 0.18 |  |  |  |  |  |
| **Hippocampus** | Baseline | 4.68 ± 0.56 | 0.4449 | **0.0485** | **0.0082** | 0.9311 | >0.9999 |
|  | Pre-onset | 4.33 ± 0.54 |  | 0.9716 | 0.9650 | 0.9430 | 0.2236 |
|  | Onset | 4.19 ± 0.37 |  |  | >0.9999 | 0.1035 | 0.2464 |
|  | Peak | 4.19 ± 0.54 |  |  |  | 0.3215 | 0.0630 |
|  | Remission | 4.52 ± 0.29 |  |  |  |  | 0.9310 |
|  | Relapse | 4.65 ± 0.27 |  |  |  |  |  |
| **Midbrain** | Baseline | 4.07 ± 0.49 | >0.9999 | 0.7854 | 0.9960 | >0.9999 | 0.9997 |
|  | Pre-onset | 4.05 ± 0.33 |  | 0.6079 | >0.9999 | >0.9999 | 0.9985 |
|  | Onset | 4.26 ± 0.35 |  |  | 0.7468 | 0.7464 | 0.8364 |
|  | Peak | 4.01 ± 0.70 |  |  |  | 0.9982 | 0.9955 |
|  | Remission | 4.07 ± 0.33 |  |  |  |  | >0.9999 |
|  | Relapse | 4.10 ± 0.24 |  |  |  |  |  |
| **Hypothalamus** | Baseline | 3.73 ± 0.25 | 0.8607 | >0.9999 | 0.5813 | 0.9409 | 0.6590 |
|  | Pre-onset | 3.62 ± 0.25 |  | 0.9084 | 0.9834 | >0.9999 | >0.9999 |
|  | Onset | 3.74 ± 0.28 |  |  | 0.5482 | 0.8509 | 0.5767 |
|  | Peak | 3.56 ± 0.29 |  |  |  | 0.8508 | 0.9537 |
|  | Remission | 3.64 ± 0.25 |  |  |  |  | 0.9994 |
|  | Relapse | 3.61 ± 0.16 |  |  |  |  |  |
| **Thalamus** | Baseline | 3.68 ± 0.58 | 0.7368 | >0.9999 | 0.4504 | 0.8709 | 0.9428 |
|  | Pre-onset | 3.47 ± 0.31 |  | 0.4255 | 0.9993 | 0.9987 | 0.9692 |
|  | Onset | 3.69 ± 0.31 |  |  | 0.4999 | 0.7763 | 0.9551 |
|  | Peak | 3.41 ± 0.55 |  |  |  | 0.9577 | 0.9329 |
|  | Remission | 3.51 ± 0.36 |  |  |  |  | 0.9993 |
|  | Relapse | 3.56 ± 0.29 |  |  |  |  |  |
| **Striatum** | Baseline | 3.54 ± 0.16 | 0.9997 | 0.9950 | 0.0658 | 0.9317 | 0.8200 |
|  | Pre-onset | 3.56 ± 0.24 |  | 0.9785 | 0.1889 | 0.8939 | 0.9087 |
|  | Onset | 3.51 ± 0.27 |  |  | 0.3794 | 0.9987 | 0.9811 |
|  | Peak | 3.38 ± 0.24 |  |  |  | 0.8678 | 0.6032 |
|  | Remission | 3.47 ± 0.23 |  |  |  |  | >0.9999 |
|  | Relapse | 3.47 ± 0.14 |  |  |  |  |  |
|  | Baseline | 4.20 ± 0.29 | >0.9999 | 0.6553 | 0.9935 | >0.9999 | 0.5694 |
|  | Pre-onset | 4.19 ± 0.24 |  | 0.5416 | 0.9969 | >0.9999 | 0.6649 |
| **Pallidum** | Onset | 4.37 ± 0.26 |  |  | 0.9485 | 0.6451 | 0.9993 |
|  | Peak | 4.26 ± 0.46 |  |  |  | 0.9958 | 0.8790 |
|  | Remission | 4.19 ± 0.34 |  |  |  |  | 0.8788 |
|  | Relapse | 4.33 ± 0.15 |  |  |  |  |  |

| **Region** | **Timepoint** | **Mean ± SD (m/s)** | **Adjusted *p* value** | | | | |
| --- | --- | --- | --- | --- | --- | --- | --- |
|  |  |  | **Pre-onset** | **Onset** | **Peak** | **Remission** | **Relapse** |
| **Caudoputamen** | Baseline | 3.62 ± 0.21 | 0.9667 | 0.9245 | **0.0074** | 0.9069 | 0.3827 |
|  | Pre-onset | 3.68 ± 0.25 |  | 0.6881 | **0.0288** | 0.6439 | 0.1913 |
|  | Onset | 3.56 ± 0.30 |  |  | 0.2569 | >0.9999 | 0.7012 |
|  | Peak | 3.38 ± 0.25 |  |  |  | 0.2005 | 0.5005 |
|  | Remission | 3.56 ± 0.18 |  |  |  |  | 0.8592 |
|  | Relapse | 3.48 ± 0.04 |  |  |  |  |  |
| **Deep Gray Matter** | Baseline | 3.45 ± 0.18 | 0.2550 | 0.1760 | **0.0029** | 0.2337 | 0.2295 |
|  | Pre-onset | 3.35 ± 0.12 |  | 0.9860 | **0.0245** | 0.9873 | 0.9955 |
|  | Onset | 3.32 ± 0.17 |  |  | 0.0512 | >0.9999 | >0.9999 |
|  | Peak | 3.18 ± 0.19 |  |  |  | 0.0712 | 0.2657 |
|  | Remission | 3.33 ± 0.11 |  |  |  |  | >0.9999 |
|  | Relapse | 3.32 ± 0.12 |  |  |  |  |  |

**Supplementary Table 2.**

Regional fluidity in the mouse brain given in loss angle (ϕ). p < 0.05 is given in bold.

| **Region** | **Timepoint** | **Mean ± SD (rad)** | **Adjusted *p* value** | | | | |
| --- | --- | --- | --- | --- | --- | --- | --- |
|  |  |  | **Pre-onset** | **Onset** | **Peak** | **Remission** | **Relapse** |
| **Whole Brain** | Baseline | 0.67 ± 0.05 | 0.7493 | 0.8029 | 0.9534 | 0.9460 | 0.8834 |
|  | Pre-onset | 0.70 ± 0.04 |  | 0.1345 | 0.2322 | 0.3697 | 0.3802 |
|  | Onset | 0.65 ± 0.05 |  |  | 0.9996 | 0.9991 | >0.9999 |
|  | Peak | 0.66 ± 0.08 |  |  |  | >0.9999 | >0.9999 |
|  | Remission | 0.66 ± 0.08 |  |  |  |  | >0.9999 |
|  | Relapse | 0.66 ± 0.04 |  |  |  |  |  |
| **Cortex** | Baseline | 0.51 ± 0.07 | 0.3980 | 0.7800 | 0.8916 | >0.9999 | 0.9959 |
|  | Pre-onset | 0.56 ± 0.07 |  | 0.9743 | 0.9359 | 0.3479 | 0.8066 |
|  | Onset | 0.54 ± 0.10 |  |  | >0.9999 | 0.8983 | 0.9502 |
|  | Peak | 0.54 ± 0.10 |  |  |  | 0.9346 | 0.9757 |
|  | Remission | 0.51 ± 0.08 |  |  |  |  | 0.9990 |
|  | Relapse | 0.52 ± 0.05 |  |  |  |  |  |
| **Hippocampus** | Baseline | 1.04 ± 0.17 | >0.9999 | **0.0070** | **0.0138** | 0.0876 | 0.6582 |
|  | Pre-onset | 1.03 ± 0.17 |  | **0.0109** | **0.0013** | **0.0473** | 0.5490 |
|  | Onset | 0.82 ± 0.14 |  |  | >0.9999 | 0.9855 | 0.2619 |
|  | Peak | 0.82 ± 0.09 |  |  |  | 0.9770 | 0.0955 |
|  | Remission | 0.85 ± 0.14 |  |  |  |  | 0.5255 |
|  | Relapse | 0.93 ± 0.04 |  |  |  |  |  |
| **Midbrain** | Baseline | 0.96 ± 0.12 | 0.4937 | 0.9998 | 0.9949 | >0.9999 | >0.9999 |
|  | Pre-onset | 0.91 ± 0.09 |  | 0.7935 | 0.9344 | 0.6499 | 0.9256 |
|  | Onset | 0.95 ± 0.09 |  |  | 0.9996 | >0.9999 | >0.9999 |
|  | Peak | 0.94 ± 0.10 |  |  |  | 0.9980 | 0.9995 |
|  | Remission | 0.95 ± 0.10 |  |  |  |  | >0.9999 |
|  | Relapse | 0.95 ± 0.11 |  |  |  |  |  |
| **Hypothalamus** | Baseline | 0.73 ± 0.08 | 0.8133 | 0.4622 | 0.3030 | 0.9997 | 0.3229 |
|  | Pre-onset | 0.76 ± 0.07 |  | 0.0537 | **0.0239** | 0.9167 | 0.1589 |
|  | Onset | 0.68 ± 0.07 |  |  | 0.9989 | 0.5604 | >0.9999 |
|  | Peak | 0.67 ± 0.08 |  |  |  | 0.2764 | 0.9988 |
|  | Remission | 0.73 ± 0.09 |  |  |  |  | 0.7307 |
|  | Relapse | 0.68 ± 0.06 |  |  |  |  |  |
| **Thalamus** | Baseline | 1.00 ± 0.05 | 0.8975 | >0.9999 | 0.9839 | 0.9996 | 0.9445 |
|  | Pre-onset | 0.98 ± 0.06 |  | 0.9116 | >0.9999 | 0.9617 | 0.5990 |
|  | Onset | 1.00 ± 0.06 |  |  | 0.8292 | >0.9999 | 0.9953 |
|  | Peak | 0.98 ± 0.08 |  |  |  | 0.7870 | 0.9295 |
|  | Remission | 1.01 ± 0.11 |  |  |  |  | 0.9999 |
|  | Relapse | 1.02 ± 0.07 |  |  |  |  |  |
| **Striatum** | Baseline | 0.78 ± 0.06 | 0.9988 | 0.2577 | **0.0086** | 0.4121 | 0.0843 |
|  | Pre-onset | 0.79 ± 0.05 |  | **0.0330** | **0.0208** | 0.6719 | 0.0898 |
|  | Onset | 0.73 ± 0.05 |  |  | 0.9789 | 0.8826 | 0.9546 |
|  | Peak | 0.72 ± 0.07 |  |  |  | 0.2630 | 0.9988 |
|  | Remission | 0.76 ± 0.07 |  |  |  |  | 0.4800 |
|  | Relapse | 0.71 ± 0.06 |  |  |  |  |  |
|  | Baseline | 0.82 ± 0.09 | 0.8824 | 0.3177 | 0.2771 | 0.8839 | 0.4048 |
|  | Pre-onset | 0.85 ± 0.08 |  | **0.0208** | **0.0469** | >0.9999 | 0.2598 |
| **Pallidum** | Onset | 0.76 ± 0.08 |  |  | 0.9998 | 0.1614 | >0.9999 |
|  | Peak | 0.75 ± 0.11 |  |  |  | 0.0931 | >0.9999 |
|  | Remission | 0.86 ± 0.12 |  |  |  |  | 0.6178 |
|  | Relapse | 0.75 ± 0.11 |  |  |  |  |  |

| **Region** | **Timepoint** | **Mean ± SD (rad)** | **Adjusted *p* value** | | | | |
| --- | --- | --- | --- | --- | --- | --- | --- |
|  |  |  | **Pre-onset** | **Onset** | **Peak** | **Remission** | **Relapse** |
| **Caudoputamen** | Baseline | 0.82 ± 0.05 | 0.6314 | 0.9295 | 0.1293 | 0.9914 | 0.2191 |
|  | Pre-onset | 0.85 ± 0.08 |  | 0.2775 | 0.0559 | 0.7796 | 0.0649 |
|  | Onset | 0.79 ± 0.08 |  |  | 0.8995 | 0.9993 | 0.7289 |
|  | Peak | 0.76 ± 0.09 |  |  |  | 0.5245 | 0.8871 |
|  | Remission | 0.80 ± 0.10 |  |  |  |  | 0.6325 |
|  | Relapse | 0.73 ± 0.08 |  |  |  |  |  |
| **Deep Gray Matter** | Baseline | 0.73 ± 0.06 | >0.9999 | 0.2051 | 0.1595 | 0.6387 | 0.2018 |
|  | Pre-onset | 0.73 ± 0.03 |  | **0.0155** | 0.0571 | 0.6642 | 0.1143 |
|  | Onset | 0.68 ± 0.04 |  |  | 0.9998 | 0.8609 | >0.9999 |
|  | Peak | 0.68 ± 0.06 |  |  |  | 0.9088 | 0.9867 |
|  | Remission | 0.70 ± 0.06 |  |  |  |  | 0.8045 |
|  | Relapse | 0.68 ± 0.04 |  |  |  |  |  |

**Supplementary Table 3.**

T2 relaxation times in the mouse brain given in milliseconds (ms). p < 0.05 is given in bold.

| **Region** | **Timepoint** | **Mean ± SD (ms)** | **Adjusted *p* value** | | | | |
| --- | --- | --- | --- | --- | --- | --- | --- |
|  |  |  | **Pre-onset** | **Onset** | **Peak** | **Remission** | **Relapse** |
| **Whole Brain** | Baseline | 48.44 ± 0.67 | 0.8563 | 0.9998 | 0,3394 | 0.9851 | 0.3768 |
|  | Pre-onset | 48.76 ± 0.46 |  | 0.1062 | **0.0155** | 0.9897 | 0.2192 |
|  | Onset | 48.37 ± 0.57 |  |  | 0.2563 | 0.9160 | 0.6648 |
|  | Peak | 47.80 ± 0.49 |  |  |  | **0.0101** | >0.9999 |
|  | Remission | 48.63 ± 0.31 |  |  |  |  | 0.0532 |
|  | Relapse | 47.79 ± 0.68 |  |  |  |  |  |
| **Cortex** | Baseline | 48.21 ± 0.81 | 0.9716 | 0.9353 | 0.2493 | 0.9411 | 0.3923 |
|  | Pre-onset | 48.48 ± 0.59 |  | 0.2370 | 0.0672 | >0.9999 | 0.2325 |
|  | Onset | 47.96 ± 0.54 |  |  | 0.5136 | 0.2592 | 0.7675 |
|  | Peak | 47.54± 0.48 |  |  |  | **0.0086** | 0.9886 |
|  | Remission | 48.53± 0.34 |  |  |  |  | 0.0556 |
|  | Relapse | 47.40± 0.76 |  |  |  |  |  |
| **Hippocampus** | Baseline | 51.17 ± 1.42 | 0.9976 | 0.9995 | 0.3735 | 0.3317 | 0.2781 |
|  | Pre-onset | 51.02 ± 1.10 |  | 0.9921 | 0.3467 | 0.1116 | 0.2410 |
|  | Onset | 51.36 ± 1.16 |  |  | 0.1266 | 0.6193 | 0.1026 |
|  | Peak | 49.97 ± 1.16 |  |  |  | 0.9966 | 0.4266 |
|  | Remission | 50.25 ± 1.26 |  |  |  |  | 0.7540 |
|  | Relapse | 48.95 ± 1.48 |  |  |  |  |  |
| **Midbrain** | Baseline | 46.59 ± 1.27 | 0.9753 | 0.4157 | 0.9998 | 0.8394 | 0.9534 |
|  | Pre-onset | 47.13 ± 1.92 |  | 0.4075 | 0.9759 | >0.9999 | >0.9999 |
|  | Onset | 47.97 ± 2.00 |  |  | 0.3340 | 0.8153 | 0.7866 |
|  | Peak | 46.72 ± 1.01 |  |  |  | 0.9934 | 0.9879 |
|  | Remission | 47.04 ± 1.12 |  |  |  |  | >0.9999 |
|  | Relapse | 47.06 ± 1.38 |  |  |  |  |  |
| **Hypothalamus** | Baseline | 48.92 ± 1.01 | 0.9966 | 0.9998 | 0.9998 | 0.8897 | 0.8271 |
|  | Pre-onset | 49.08 ± 0.71 |  | 0.9991 | 0.9918 | 0.9960 | 0.9783 |
|  | Onset | 49.01± 0.62 |  |  | 0.9951 | 0.9539 | 0.9453 |
|  | Peak | 48.82 ± 0.93 |  |  |  | 0.6344 | 0.6080 |
|  | Remission | 49.28 ± 0.79 |  |  |  |  | 0.9991 |
|  | Relapse | 49.35 ± 0.70 |  |  |  |  |  |
| **Thalamus** | Baseline | 46.10 ± 0.55 | 0.3928 | 0.4653 | 0.9805 | 0.9995 | 0.7777 |
|  | Pre-onset | 46.70 ± 0.91 |  | 0.9996 | 0.4724 | 0.6567 | 0.7286 |
|  | Onset | 46.84 ± 1.29 |  |  | 0.4238 | 0.7273 | 0.4752 |
|  | Peak | 45.90 ± 0.96 |  |  |  | 0.9996 | 0.9999 |
|  | Remission | 46.03 ± 0.87 |  |  |  |  | 0.9409 |
|  | Relapse | 45.82 ± 0.10 |  |  |  |  |  |
| **Striatum** | Baseline | 49.12 ± 0.89 | 0.9876 | 0.9443 | 0.6860 | >0.9999 | 0.5359 |
|  | Pre-onset | 49.36 ± 0.50 |  | 0.3892 | 0.1450 | 0.8263 | 0.1980 |
|  | Onset | 48.62 ± 1.19 |  |  | 0.9109 | 0.7511 | 0.9939 |
|  | Peak | 48.24 ± 1.19 |  |  |  | 0.1861 | 0.9989 |
|  | Remission | 49.19 ± 0.53 |  |  |  |  | 0.1476 |
|  | Relapse | 48.42 ± 0.75 |  |  |  |  |  |
|  | Baseline | 46.49 ± 0.87 | 0.8617 | 0,5762 | >0.9999 | 0.9936 | 0.8342 |
|  | Pre-onset | 46.91 ± 0.79 |  | 0.9208 | 0.8841 | 0.9834 | 0.4334 |
| **Pallidum** | Onset | 47.15 ± 0.60 |  |  | 0.3423 | 0.6461 | **0.0191** |
|  | Peak | 46.50 ± 0.62 |  |  |  | 0.9828 | 0.5598 |
|  | Remission | 46.64 ± 0.72 |  |  |  |  | 0.4741 |
|  | Relapse | 46.03 ± 0.71 |  |  |  |  |  |

| **Region** | **Timepoint** | **Mean ± SD (ms)** | **Adjusted *p* value** | | | | |
| --- | --- | --- | --- | --- | --- | --- | --- |
|  |  |  | **Pre-onset** | **Onset** | **Peak** | **Remission** | **Relapse** |
| **Caudoputamen** | Baseline | 47.91 ± 0.80 | 0.9962 | 0.6653 | 0.1503 | 0.9999 | 0.2546 |
|  | Pre-onset | 48.07 ± 0,48 |  | 0.0836 | **0.0056** | 0.5439 | 0.1009 |
|  | Onset | 47.11 ± 1.13 |  |  | 0.5557 | 0.5491 | 0.9845 |
|  | Peak | 46.57± 0.93 |  |  |  | **0.0194** | 0.9686 |
|  | Remission | 47.81± 0.71 |  |  |  |  | 0.1689 |
|  | Relapse | 46.84 ± 0.69 |  |  |  |  |  |
| **Deep Gray Matter** | Baseline | 50.79 ± 0.76 | 0.9891 | 0.9546 | 0.9816 | 0.6683 | 0.9647 |
|  | Pre-onset | 50.97 ± 0.49 |  | 0.9955 | 0.5005 | 0.9493 | 0.6725 |
|  | Onset | 51.05 ± 0.60 |  |  | 0.5494 | 0.9935 | 0.6287 |
|  | Peak | 50.54 ± 0.56 |  |  |  | 0.0821 | 0.9994 |
|  | Remission | 51.20 ± 0.36 |  |  |  |  | 0.1589 |
|  | Relapse | 50.59 ± 0.56 |  |  |  |  |  |

**Supplementary Table 4.**

Apparent diffusion coefficient (ADC) in the mouse brain given in ×10^-6^ mm^2^/s. p < 0.05 is given in bold.

| **Region** | **Timepoint** | **Mean ± SD (×10^-6^ mm^2^/s)** | **Adjusted *p* value** | | | | |
| --- | --- | --- | --- | --- | --- | --- | --- |
|  |  |  | **Pre-onset** | **Onset** | **Peak** | **Remission** | **Relapse** |
| **Whole Brain** | Baseline | 797.5 ± 103.0 | 0.9622 | >0.9999 | 0.4679 | 0.9788 | 0.9979 |
|  | Pre-onset | 755.5 ± 82.04 |  | 0.9639 | 0.7385 | 0.4648 | >0.9999 |
|  | Onset | 813.0 ± 171.8 |  |  | 0.5742 | 0.9987 | 0.9933 |
|  | Peak | 697.0 ± 46.96 |  |  |  | **0.0355** | 0.8529 |
|  | Remission | 841.2 ± 105.0 |  |  |  |  | 0.7456 |
|  | Relapse | 760.5 ± 103.0 |  |  |  |  |  |
| **Cortex** | Baseline | 794.1 ± 118.5 | 0.9945 | >0.9999 | 0.4289 | 0.9835 | 0.9970 |
|  | Pre-onset | 762.1 ± 96.22 |  | 0.9987 | 0.4987 | 0.6754 | 0.9999 |
|  | Onset | 786.1 ± 150.6 |  |  | 0.4876 | 0.9731 | 0.9981 |
|  | Peak | 671.7 ± 51.34 |  |  |  | 0.0566 | 0.6264 |
|  | Remission | 839.5 ± 125.0 |  |  |  |  | 0.6657 |
|  | Relapse | 751.5 ± 92.91 |  |  |  |  |  |
| **Hippocampus** | Baseline | 898.5 ± 162.6 | 0.9976 | 0.9995 | 0.3735 | 0.3317 | 0.2781 |
|  | Pre-onset | 763.5 ± 114.9 |  | 0.9921 | 0.3467 | 0.1166 | 0.2410 |
|  | Onset | 804.1 ± 181.1 |  |  | 0.1266 | 0.6193 | 0.1026 |
|  | Peak | 766.2 ± 88.49 |  |  |  | 0.9966 | 0.4266 |
|  | Remission | 873.4 ± 74.14 |  |  |  |  | 0.7540 |
|  | Relapse | 738.9 ± 72.28 |  |  |  |  |  |
| **Midbrain** | Baseline | 839.3 ± 187.4 | 0.5093 | 0.9694 | 0.9586 | 0.7662 | 0.6345 |
|  | Pre-onset | 707.6 ± 133.4 |  | 0.5790 | 0.8546 | 0.8369 | >0.9999 |
|  | Onset | 777.3 ± 95.04 |  |  | >0.9999 | 0.9993 | 0.8437 |
|  | Peak | 774.2 ± 84.20 |  |  |  | 0.9978 | 0.8428 |
|  | Remission | 790.1 ± 105.1 |  |  |  |  | 0.7715 |
|  | Relapse | 715.4 ± 50.64 |  |  |  |  |  |
| **Hypothalamus** | Baseline | 781.8 ± 90.60 | 0.9938 | 0.9441 | 0.6232 | 0.7993 | 0.9971 |
|  | Pre-onset | 756.2 ± 109.0 |  | 0.8751 | 0.9704 | 0.3725 | 0.9999 |
|  | Onset | 859.3 ± 195.9 |  |  | 0.5356 | >0.9999 | 0.9089 |
|  | Peak | 716.7 ± 44.14 |  |  |  | **0.0215** | 0.9979 |
|  | Remission | 857.4 ± 110.3 |  |  |  |  | 0.5322 |
|  | Relapse | 743.3 ± 119.8 |  |  |  |  |  |
| **Thalamus** | Baseline | 802.1 ± 127.9 | 0.4869 | 0.9991 | 0.6775 | 0.9923 | >0.9999 |
|  | Pre-onset | 716.5 ± 58.81 |  | 0.8129 | >0.9999 | 0.1552 | 0.8683 |
|  | Onset | 841.8 ± 241.2 |  |  | 0.7420 | >0.9999 | 0.9964 |
|  | Peak | 712.6 ± 43.86 |  |  |  | 0.0602 | 0.8859 |
|  | Remission | 842.4 ± 104.5 |  |  |  |  | 0.7729 |
|  | Relapse | 782.2 ± 120.9 |  |  |  |  |  |
| **Striatum** | Baseline | 711.3 ± 59.71 | 0.9611 | 0.9656 | 0.5918 | 0.7912 | 0.9986 |
|  | Pre-onset | 764.3 ± 135.9 |  | >0.9999 | 0.4037 | 0.9993 | 0.9996 |
|  | Onset | 760.8 ± 158.1 |  |  | 0.5093 | 0.9992 | >0.9999 |
|  | Peak | 650.5 ± 60.43 |  |  |  | 0.2376 | 0.7919 |
|  | Remission | 784.5 ± 113.7 |  |  |  |  | 0.9922 |
|  | Relapse | 742.7 ± 170.2 |  |  |  |  |  |
|  | Baseline | 712.6 ± 58.43 | 0.9915 | 0.7305 | 0.9829 | 0.5351 | 0.7717 |
|  | Pre-onset | 745.7 ± 127.5 |  | 0.9346 | 0.8626 | 0.9283 | 0.9460 |
| **Pallidum** | Onset | 837.7 ± 230.2 |  |  | 0.6144 | >0.9999 | 0.9998 |
|  | Peak | 693.7 ± 49.71 |  |  |  | 0.3061 | 0.6829 |
|  | Remission | 820.9 ± 125.9 |  |  |  |  | >0.9999 |
|  | Relapse | 799.7 ± 136.1 |  |  |  |  |  |

| **Region** | **Timepoint** | **Mean ± SD (×10^-6^ mm^2^/s)** | **Adjusted *p* value** | | | | |
| --- | --- | --- | --- | --- | --- | --- | --- |
|  |  |  | **Pre-onset** | **Onset** | **Peak** | **Remission** | **Relapse** |
| **Caudoputamen** | Baseline | 699.4 ± 63.86 | 0.9221 | 0.9990 | 0.4956 | 0.8776 | 0.9992 |
|  | Pre-onset | 766.0 ± 142.8 |  | 0.9621 | 0.2663 | >0.9999 | 0.9890 |
|  | Onset | 717.9 ± 125.9 |  |  | 0.5016 | 0.9843 | >0.9999 |
|  | Peak | 626.9 ± 63.93 |  |  |  | 0.2684 | 0.8364 |
|  | Remission | 760.9 ± 114.8 |  |  |  |  | 0.9812 |
|  | Relapse | 724.0 ± 162.5 |  |  |  |  |  |
| **Deep Gray Matter** | Baseline | 785.6 ± 81.37 | 0.9891 | 0.9546 | 0.9816 | 0.6683 | 0.9647 |
|  | Pre-onset | 757.6 ± 88.52 |  | 0.9955 | 0.5005 | 0.9493 | 0.6725 |
|  | Onset | 787.8 ± 153.1 |  |  | 0.5494 | 0.9935 | 0.6287 |
|  | Peak | 700.1 ± 41.25 |  |  |  | 0.0821 | 0.9994 |
|  | Remission | 829.3 ± 88.6 |  |  |  |  | 0.1589 |
|  | Relapse | 758. 6 ± 69.73 |  |  |  |  |  |
